# Supplementary material for: High-throughput telomere length measurement at nucleotide resolution using the PacBio high fidelity sequencing platform
Source: Nat Commun. 2023 Jan 17;14:281. doi: 10.1038/s41467-023-35823-7 (PMC9845338; doi:10.1038/s41467-023-35823-7)
Supplement: Supplementary file 1 — Editorial Assessment Report [file 41467_2023_35823_MOESM1_ESM.pdf]

nature portfolio

GUIDED OPEN ACCESS

nature methods | nature communications | communications biology

Contents of this report

1. Manuscript details: overview of your manuscript and the editorial team.
2. Review synthesis: summary of the reviewer reports provided by the editors.
3. Editorial recommendation: personalized evaluation and recommendation from all 3 journals.
4. Annotated reviewer comments: the referee reports with comments from the editors.
5. Open research evaluation: advice for adhering to best reproducibility practices.

About the editorial process

Because you selected the **Nature Portfolio Guided Open Access** option, your manuscript was assessed for suitability in three of our titles publishing high-quality work across the spectrum of methods research: ***Nature Methods*, *Nature Communications*, and *Communications Biology***. More information about Guided Open Access can be found here.

Collaborative editorial assessment

Your editorial team discussed the manuscript to determine its suitability for the Nature Portfolio Guided OA pilot. Our assessment of your manuscript takes into account several factors, including whether the work meets the **technical standard** of the Nature Portfolio and whether the findings are of **immediate significance** to the readership of at least one of the participating journals in the Nature Portfolio Guided Open Access methods cluster.

Peer review

Experts were asked to evaluate the following aspects of your manuscript:

- **Novelty** in comparison to prior publications;
- **Likely audience** of researchers in terms of broad fields of study and size;
- **Potential impact** of the study on the immediate or wider research field;
- **Evidence** for the claims and whether additional experiments or analyses could feasibly strengthen the evidence;
- **Methodological detail** and whether the manuscript is reproducible as written;
- Appropriateness of the **literature review**.

Editorial evaluation of reviews

Your editorial team discussed the potential suitability of your manuscript for each of the participating journals. They then discussed the revisions necessary in order for the work to be published, keeping each journal's specific editorial criteria in mind.

Journals in the Nature portfolio will support authors wishing to transfer their reviews and (where reviewers agree) the reviewers' identities to journals outside of Springer Nature.
If you have any questions about review portability, please contact our editorial office at guidedoa@nature.com.

nature portfolio

GUIDED OPEN ACCESS
nature methods | nature communications | communications biology

Manuscript details

Tracking number	Submission date	Decision date	Peer review type
GUIDEDOA-22-00394	**Jan 17, 2022**	**Mar 7, 2022**	Single-blind

Manuscript title

High-throughput telomere length measurement at nucleotide resolution using the PacBio high fidelity sequencing platform

Author details

Shang Li

Affiliation: Duke-NUS Medical School

Editorial assessment team

**Primary editor**	**George Inglis**<br>Home journal: ***Communications Biology***<br>ORCID: **0000-0002-9069-5242**<br>Email: **george.inglis@us.nature.com**
**Other editors consulted**	**Lin Tang**<br>Home journal: ***Nature Methods***<br>ORCID: **0000-0002-6050-0424**<br><br>**Cara Eldrige**<br>Home journal: ***Nature Communications***<br>ORCID: **0000-0001-7001-2312**
**About your primary editor**	George received his PhD in Genetics and Molecular Biology from Emory University, where he studied mouse models of voltage-gated sodium channel dysfunction and epilepsy. He also has research experience in epigenomics and *in vitro* models of neuronal development. George joined the editorial team of *Communications Biology* in September 2020 and is based in the New York office.

nature portfolio

GUIDED OPEN ACCESS
nature methods · nature communications · communications biology

Editorial assessment and review synthesis

**Editor's summary and assessment**	Here, the authors present a workflow to measure telomere length at single-telomere resolution. This approach relies on using biotinylated probes (termed, "telobaits") that are complementary to G-rich overhangs at 3' ends of chromosomes, allowing users to purify full-length telomeres from gDNA fragments, which are then put through PacBio/SMRT long-read sequencing. Since each probe has its own barcode, they can therefore identify the lengths of individual telomeres, and structural variants within telomeres. They use this methodology to probe telomere lengths in 5 immortalized cell lines (HCT116, HEK293T, T24, IMR90, and WI38) and PBMCs from 48 human participants (as a demonstration of high-throughput application), as well as a control telomere. The telomere characteristics of these cells are consistent with previous reports, suggesting the validity of this method and its future utility in characterizing the relationships between telomere length in human aging and disease.<br><br>While the editors jointly decided to send this manuscript out to review based on the value of probing single-telomere lengths, there were some concerns about the conceptual advance over existing approaches that prohibited further consideration by *Nature Methods*.
**Editorial synthesis of reviewer reports**	Both reviewers comment on the potential utility of this method, and suggest a few more analyses to fine-tune the conclusions. Reviewer #1 outlines the need for additional data (including diverse cohorts) to support any claims of telomere fingerprinting and points out a potential artifact for short (12 nt) reads. Reviewer #2 asks for more clarity on sex- or chromosome-specific results (among other minor discussion points), and reiterates the need from Reviewer #1 for more detail in the Methods section.<br><br>In light of this positive feedback, *Nature Communications* would also be interested in seeing a major revision with extension of the work to address the concerns raised by Reviewer #1.<br><br>*Communications Biology* would also be interested in considering a revision that explicitly states the limitations outlined by Reviewer #1 (and qualifies any claims of fingerprinting), expands the methods and discussion of literature as suggested by both reviewers, and addresses potential artifacts and other minor analyses suggested by both reviewers.

Editorial recommendation

Nature Methods<br><br>Revision not invited	The conceptual advance is not sufficient for publication in *Nature Methods*.
Nature Communications<br><br>Major revisions with extension of the work	*Nature Communications* would be happy to consider a revised manuscript with additional data to support your conclusions, as suggested by Reviewer #1. However, we understand that the extension of your work is not trivial, so please see below for information on continuing the review process with *Communications Biology*.
Communications Biology<br><br>Minor revisions	*Communications Biology* would be interested in considering a revision that explicitly states the limitations outlined by Reviewer #1 (and qualifies any claims of fingerprinting), expands the methods and discussion of literature as suggested by both reviewers, and addresses potential artifacts and other minor analyses suggested by both reviewers.

Next steps

**Editorial recommendation 1:**	Our top recommendation is to revise and resubmit your manuscript to *Nature Communications.* We feel the additional experiments required are reasonable to address within a 6-month time frame.
**Editorial recommendation 2:**	You may also choose to revise and resubmit your manuscript to *Communications Biology.* This option might be best if the requested experimental revisions are not possible/feasible at this time.
**Note**	As stated on the previous page *Nature Methods* is not inviting a revision at this time. Please keep in mind that the journal will not be able to consider any appeals of their decision through Guided Open Access.

Revision

To follow our recommendation, please upload the revised manuscript files using **the link provided in the decision letter**. Should you need assistance with our manuscript tracking system, please contact Adam Lipkin, our Nature Portfolio Guided OA support specialist, at guidedOA@nature.com.

Revision checklist

- ☐ Cover letter, stating to which journal you are submitting
- ☐ Revised manuscript
- ☐ Point-by-point response to reviews
- ☐ Updated Reporting Summary and Editorial Policy Checklist
- ☐ Supplementary materials (if applicable)

Submission elsewhere

If you choose not to follow our recommendations, you can still take the reviewer reports with you.

Option 1: Transfer to another Nature Portfolio journal
Springer Nature provides authors with the ability to transfer a manuscript within the Nature Portfolio, without the author having to upload the manuscript data again. To use this service, **please follow the transfer link provided in the decision letter.** If no link was provided, please contact guidedOA@nature.com.

> *Note that any decision to opt in to In Review at the original journal is not sent to the receiving journal on transfer. You can opt in to In Review at receiving journals that support this service by choosing to modify your manuscript on transfer.*

Option 2: Portable Peer Review option for submission to a journal outside of Nature Portfolio
If you choose to submit your revised manuscript to a journal at another publisher, we can share the reviews with another journal outside of the Nature Portfolio if requested. You will need to request that the receiving journal office contacts us at guidedOA@nature.com. We have included editorial guidance below in the reviewer reports and open research evaluation to aid in revising the manuscript for publication elsewhere.

Annotated reviewer reports

The editors have included some additional comments on specific points raised by the reviewers below, to clarify requirements for publication in the recommended journal(s). However, please note that all points should be addressed in a revision, even if an editor has not specifically commented on them.

Reviewer #1 information

**Expertise**	This reviewer has expertise in long-read sequencing and gene regulation.
**Editor's comments**	This reviewer finds the method to be of some interest to the field, but raises valid concerns about its ability to "fingerprint" samples in the absence of additional, diverse cohorts. They also note a potential artifact in shorter (12 nt) reads that should be addressed, and highlight the need for more clarity throughout the Methods. Given the current limitations and conceptual advance, *Nature Methods* is unable to invite a revision.

Reviewer #1 comments

Section	Annotated Reviewer Comments
**Remarks to the Author: Overall significance**	The study by Tham et al., presents a high-throughput method for obtaining accurate telomere length measurements at single-nucleotide resolution. By leveraging highly accurate single-molecule PCR-free sequencing, this method overcomes many of the limitations associated with prior methods for determining telomere lengths, and the enrichment strategy appears well suited for multiplexing multiple samples in the same run. Overall, the method and analyses regarding telomeric variant sequences (TVSs) significantly advance the field and will likely be of general interest. However, the current manuscript contains several conclusions that are not well supported by their data, and additional discussion is needed regarding the limitations of this approach in terms of recovering accurate telomere lengths and telomere lengths specific to a given genomic location.<br><br>**Major comments:**<br>The abstract claims that "the unique distribution pattern and sequence of TVSs in individuals may be used as a fingerprint for sample identification." This is a bold claim with wide ranging implications across telomere biology, genetics, and forensics, and has the potential to be picked up in the popular press. However, no data is presented in the manuscript that substantiates this claim. Specifically, although the authors present data that TVSs overall appear to be different across the 48 patient PBMC samples, they do not actually show that if one were to repeat this method with a biological replicate of one of these samples, that it would uniquely match to the same sample. Similarly, as this study is limited to only 48 individuals, it is unclear if the authors are merely identifying various telomere haplotypes that are

specific to certain populations, or genetic features that are truly unique to each individual. Please adjust this language accordingly, or substantiate this claim by providing reproducible population-level data across biological replicates from diverse cohorts that accounts for haplotype-specific features.

Nature Communications would ask that you substantiate this claim with further data. A revision for _Communications Biology_ should remove / carefully qualify any conclusions related to sample fingerprinting, or state this point as a future direction of the study.

The abstract claims that "the presence of TVSs disrupts the continuity of the canonical (5'-TTAGGG-3')n telomere repeats, which affects the binding of shelterin complexes at the chromosomal ends and telomere protection." However, the authors do not actually demonstrate that TVSs disrupt the binding of shelterin complexes at the chromosomal ends or disrupt telomere protection. Rather, the authors show that TVSs disrupt canonical shelterin binding elements. However, the impact of this on shelterin occupancy and telomere protection is not evaluated in this manuscript.

Nature Communications would require further experiments to explicitly show the role of TVSs in disrupting shelterin complexes. As above, a revision for _Communications Biology_ should remove / carefully qualify any conclusions related to shelterin complex protection.

The calculation of "telomere length" presented in figure 2A is a little awkward and not entirely consistent with standard definitions of telomere length. Specifically, telomere length is often thought of as the continuous linear length of DNA sequence from the start of the TTAGGG repetitive element to the terminal end of a telomere. It is unclear why the authors have chosen to use a non-continuous length measurement. It would be useful for the authors to discuss how their results could differ if they used a continuous linear length definition of telomere length (i.e., "raw telomere length").

Please justify the calculation of telomere length, for further consideration at _Nature Communications_ and _Communications Biology_.

Although this method provides "telomere length measurement at nucleotide resolution", the authors have not demonstrated that this method can substantially determine which specific telomere a given read arises from. Specifically, the authors only report genomic-location specific results for 15q and 19q (figure 5B), with all other analyses being agnostic of specific genomic location. I suspect that this is at least partially due to the use of hg38 as the reference genome, as telomeric regions within hg38 are quite problematic. However, the lack of genomic position-precise maps beyond 15q and 19q limits the ability to determine the true extent of TVSs within an individual, as it is unclear based on the findings whether these TVSs are fixed within an individual on a given telomere end or are variable across different cells from the same individual. This latter point would have significant implications for using TVSs as a "fingerprinting method".

Nature Communications would ask that you address the issue of whether

TVSs are fixed within an individual on a given telomere end or are variable across different cells from the same individual. Please explicitly state this point as a limitation, for further consideration at *Communications Biology*.

For the reads with only a 12nt telomere length, can the authors address whether the ends of these reads happen to correspond to digestion sites for RsaI, HinfI or EcoRI within TVSs, as this would indicate a potential artifactual mechanism for these reads? Similarly, do these reads from the same individual have similar sub-telomeric sequences, which would suggest an off target restriction digestion issue as opposed to a shearing issue as is proposed by the authors.

This point would be necessary for further consideration at *Nature Communications* and *Communications Biology*.

Minor comments:
The analysis of G-strand ends does not account for the contribution of potential differences in the ligation efficiencies of the 6 telobaits.

If available, the ethnicity of the 48 individuals should be reported to aid in future studies integrating this data.

Please add additional sequencing details such as:

1. Number of ZMWs with sequence information from the 48-sample multiplexed run (based on figure 3A, it appears that only ~250,000 reads were recovered, was the SMRT cell underloaded, or were there a lot of reads that failed to get CCS coverage?);
2. Per-read CCS coverage versus CCS read length plot from the aforementioned run;
3. Demultiplexing efficiency from the aforementioned run (i.e., how many reads were demultiplexed versus those that were unable to be demultiplexed).

   For the sake of reproducibility, please elaborate on these details in the Methods for further consideration at *Communications Biology* and *Nature Communications*. Please also review the Open Research Evaluation at the end of this document for other suggestions to improve transparency and data reporting.

Figure S1C, what is the etiology of those three spikes in the histogram on the right?

Please indicate whether the cell lines used in this manuscript utilize telomerase or ALT.

Please discuss whether this method would work in cells that utilize ALT.

Please include in the methods the type of blood collection tubes used.

Reviewer #2 information

**Expertise**	This reviewer has expertise in variant annotation and computational genomics.
**Editor's comments**	This reviewer provides a positive outlook on this method, and suggests several useful discussion points and minor analyses that should be incorporated in a revision.

Reviewer #2 comments

Section	Annotated Reviewer Comments
**Remarks to the Author: Overall significance**	In this paper, "High-throughput telomere length measurement at nucleotide resolution using the PacBio high fidelity sequencing platform," Tham, Poon, and colleagues used "telobaits" complementary to the single-stranded G-rich 3' telomeric overhangs, which they used to capture full-length telomeres and then use single-molecule real-time (SMRT) sequencing.<br><br>They claim their new method is a valuable tool for population-wide epidemiology studies as well as for the longitudinal assessment of telomere attrition in individuals. They also show extreme heterogeneity of telomeric variant sequences (TVSs) dispersed throughout the telomere repeat region, including a unique distribution pattern and sequence of TVSs in individuals that may be used as a fingerprint for sample identification. Finally, they provide evidence that TVSs disrupt the continuity of the canonical (5'-TTAGGG-3')n telomere repeats, which affects the binding of shelterin complexes at the chromosomal ends and telomere protection.<br><br>Overall, the work is intriguing and opens new directions in research, and the method is well-validated and useful, thus I would only request although some parts of the text need clarification or updating:<br><br>1) The authors state that previous work has shown that "telomeric repeat regions are interspersed with variant sequences exhibiting extreme heterogenicity, which may arise from errors during the sequencing of such repetitive DNA loci." However, as they and the previous paper showed, this is not a concern with HiFi reads, so I would change this to say "which may be related to sequencing kinetics in repetitive areas," since that is really the question.<br><br>2) The authors noted that they saw "slight variation in mean observed between independent sequencing runs might be due to varying loading efficiencies, as a loading bias is known to exist for shorter genomic DNA fragments." But, if they

	normalized each plot by the loading efficiency, could this issue be corrected?<br><br>3) They found a indicates a negative correlation with r=-0.447 and p-value=0.001 for the telomere length and age, but was this better on some chromosomes vs. others?<br><br>4) When they ran their PBMCs to look at telomeres, were there any differences between males and females?<br><br>5) Related to #4, were any non-canonical motifs apparently unique to one person, vs. those which were more common?<br>**Please incorporate the analyses suggested in points #2-5 for further consideration at *Nature Communications* and *Communications Biology*.**<br><br>6) Some other literature has shown that telomere length and sequence mapping can also occur on Oxford Nanopore Technologies and show variation in others contexts, and this should be referenced as well: https://www.cell.com/cell-reports/fulltext/S2211-1247(20)31446-7 and https://www.cell.com/cell-reports/fulltext/S2211-1247(20)31424-8<br>**For the sake of context, please incorporate these references into the Discussion or Introduction.**
**Remarks to the Author: Impact**	*Nature Methods* perhaps.<br>**While we appreciate the reviewer's input, all decisions regarding publication are solely made by editors.**
**Remarks to the Author: Strength of the claims**	Yes it looks good
**Remarks to the Author: Reproducibility**	Need to see the methods and raw data posted - that was not clear.<br>**This point was also raised by Reviewer #1.**

Open research evaluation

Guidelines for Transparency and Openness Promotion (TOP) in Journal Policies and Practices (“TOP Guidelines”)

The recommendations and requests in the table below are aimed at bringing your manuscript in line with common community standards as exemplified by the TOP Guidelines. While every publisher and journal will implement these guidelines differently, the recommendations below are all consistent with the policies at Nature Portfolio. In most cases, these will align with TOP Guidelines Level 2.

FAIR Principles

The goal of the recommendations in the table below related to **data or code** availability is to promote the FAIR Guiding Principles for scientific data management and stewardship (*Scientific Data* **3**: 160018, 2016). The FAIR Principles are a set of guidelines for improving 4 important aspects of digital research objects: **F**indability, **A**ccessibility, **I**nteroperability and **R**eusability.

ORCID

ORCID is a non-profit organization that provides researchers with a unique digital identifier. These identifiers can be used by editors, funding agencies, publishers, and institutions to reliably identify individuals in the same way that ISBNs and DOIs identify books and articles. Thus the risk of confusing your identity with another researcher with the same name is eliminated. **The ORCID website** provides researchers with a page where your comprehensive research activity can be stored.

Springer Nature collaborates with the ORCID organization to ensure that your research contributions (as authors and peer reviewers) are correctly attributed to you. Learn more at https://www.springernature.com/gp/researchers/orcid

Data availability

Data Availability Statement

Thank you for including a Data Availability statement. While you have included some important information, the editors have noted that some details appear to be missing.The Data Availability Statement should be as detailed as possible and include accession codes or other unique IDs for deposited data, information about where source data can be found, and specify any restrictions to data access that may apply. At a minimum, the statement should indicate that data are available upon request and explain how data access can be granted. If data access is not possible, the reasons for this must be made clear in the Data Availability Statement.

More information about the Nature Portfolio data availability policy can be found here:

More information about formatting Data Availability Statements can be found here:

Please clarify in the Data Availability statement where source data underlying Fig 2c, 2e, 3c-d, 4, and 5c can be obtained.

Mandatory data deposition

Most scientific journals, including all Nature Portfolio journals, require that any newly-generated DNA sequence data must be made publicly available before publication. There are some exceptions allowed for sensitive clinical data, but this should be discussed with the editor. All data must be deposited in a community-approved repository and accession codes/unique IDs must be included within the Data Availability Statement in the manuscript.

Examples of appropriate public repositories are listed below:

- GenBank
- Sequence Read Archive (WGS or WES data)
- The European Nucleotide Archive (ENA)

More information on mandatory data deposition policies at the Nature Portfolio can be found at http://www.nature.com/authors/policies/availability.html#data

Please visit this link for a list of approved repositories for various data types.

Other data requests

All source data underlying the graphs and charts presented in the main figures must be made available as Supplementary Data (in Excel or text format) or via a generalist repository (eg, Figshare or Dryad). This is mandatory for publication in a Nature Portfolio journal, but is also best practice for publication in any venue.

The following figures require associated source data: Fig 2c, 2e, 3c-d, 4, and 5c

Data citation
Please cite (within the main reference list) any datasets stored in external repositories that are mentioned within their manuscript. For previously published datasets, we ask that you cite both the related research article(s) and the datasets themselves. For more information on how to cite datasets in submitted manuscripts, please see our data availability statements and data citations policy:<br><br>Citing and referencing data in publications supports reproducible research, by increasing the transparency and provenance tracking of data generated or analysed during research. Citing data formally in reference lists also helps facilitate the tracking of data reuse and may help assign credit for individuals' contributions to research. A number of Springer Nature imprints are signatories of the Joint Declaration on Data Citation Principles, which stress the importance of data resources in scientific communication.

Code availability and citation
To adhere to community standards and promote transparency in research, any custom software or code should be made publicly available, ideally before publication so that referees can test the code and comment on it.<br><br>Please include a statement under the heading "Code Availability", indicating whether and how the custom code/software reported in your study can be accessed, including any restrictions to access. This section should also include information on the versions of any software used, if relevant, and any specific variables or parameters used to generate, test, or process the current dataset. Code availability statements should be provided as a separate section after the Data Availability section.<br><br>Upon publication, Nature Portfolio journals consider it best practice to release custom computer code in a way that allows readers to repeat the published results. **Code should be deposited in a DOI-minting repository such as Zenodo, Gigantum or Code Ocean and cited in the reference list** following the guidelines described in our policy pages (see link below). Authors are encouraged to manage subsequent code versions and to use a license approved by the open source initiative. Full details about how the code can be accessed and any restrictions must be described in the Code Availability statement.<br><br>See here for more information about Nature Portfolio's code availability policies: https://www.nature.com/nature-portfolio/editorial-policies/reporting-standards#availability-of-computer-code<br><br>We also provide a Code and Software submission checklist that you may find useful: https://www.nature.com/documents/nr-software-policy.pdf<br><br>Please note: because of advanced features used in this form, you must use Adobe Reader to open the document and complete it.

Ethics

We believe that authors, peer reviewers and editors should be required to disclose any competing interests that might influence their decisions and conclusions around a particular piece of content. In the interests of transparency and to help readers form their own judgements of potential bias, Nature Portfolio journals require authors to declare any competing financial and/or non-financial interests in relation to the work described.

Please provide a 'Competing interests' statement using one of the following standard sentences:

1. The authors declare the following competing interests: [specify competing interests]
2. The authors declare no competing interests.

See the Nature Portfolio competing interests policy for further information:
https://www.nature.com/nature-research/editorial-policies/competing-interests

The Springer Nature policy can be found here:
https://www.springernature.com/gp/policies/editorial-policies

We believe that research that involves the use of clinical, biomedical or biometric data from human participants must only be carried out with the explicit consent of those whose data are involved. Consent must be obtained without any form of coercion and with participants' explicit understanding of the purpose for which their data will be used.

Because your study includes human participants, confirmation that all relevant ethical regulations were followed is needed for publication in any Springer Nature journal, and that informed consent was obtained. This must be stated in the Methods section, including the name of the board and institution that approved the study protocol.

Further details about the Nature Portfolio policy can be found at
https://www.nature.com/commsbio/editorial-policies/ethics-and-biosecurity

Reporting & reproducibility

All life science papers published in Nature Portfolio journals require submission of unprocessed original images of gels and western blots to be submitted with the final accepted version in order to promote data transparency. These unprocessed images are published in the Supplementary Information.

Please include the full, uncropped blot/gel images for Fig 1b and 2f as Supplementary Figures (complete with titles and legends) and cite the new Supplementary Figures in the main manuscript text.

For more information about our image integrity policies, see
https://www.nature.com/commsbio/editorial-policies/image-integrity#electrophoretic-gels-and-blots

Cell line misidentification and cross-contamination is a
common problem with serious consequences. Authors are asked to report on the source and authentication of their cell lines.

We believe that research publications should adhere to high standards of transparency and robustness in their methods and results. This, in turn, supports the principle of reproducibility, which is a foundation of good research, especially in the natural sciences.

The Methods section should contain sufficient detail such that the work could be repeated. It is preferable that all key methods be included in the main manuscript, rather than in the Supplementary Information. Please avoid use of “as described previously” or similar, and instead detail the specific methods used, with appropriate attribution.

- **Please expand on the Southern blot was hybridization protocol**

Please note that Nature Portfolio journals allow unlimited space for Methods.

Materials availability

We recommend that you deposit any newly generated plasmids in a community repository, such as AddGene, to support open research efforts.

Oligo sequences, concentrations of antibodies, and sources of cell lines must be included in the Methods (these can also be provided in a main Table and cited in the Methods). Please see the Nature Portfolio policy page for further details.

Statistical reporting

Wherever statistics have been derived (e.g. error bars, box plots, statistical significance) figure legends should provide and define the n number (i.e. the sample size used to derive statistics) as a precise value (not a range), using the wording “n=X biologically independent samples/animals/cells/independent experiments/n= X cells examined over Y independent experiments” etc. as applicable. The figure legends must also indicate the statistical test used. Where appropriate, please indicate in the figure legends whether the statistical tests were one-sided or two-sided and whether adjustments were made for multiple comparisons. For null hypothesis testing, please indicate the test statistic (e.g. F, t, r) with confidence intervals, effect sizes, degrees of freedom and P values noted.

All error bars need to be defined in the figure legends (e.g. SD, SEM) together with a measure of centre (e.g. mean, median). For example, the legends should state something along the lines of “Data are presented as mean values +/- SEM” as appropriate. All box plots need to be defined in the legends in terms of minima, maxima, centre, bounds of box and whiskers and percentile.

For examples of expected description of statistics in figure legends, please see the following:
https://www.nature.com/articles/s41467-019-11636-5 or
https://www.nature.com/articles/s41467-019-11510-4.

When describing results as "significant" in the main text, please include details about the statistical test used and provide an exact p-value, rather than a significance threshold.
To improve reproducibility of your analyses, please provide details regarding your treatment of outliers.
To improve reproducibility of your analyses, please detail the methods used for data fitting and provide a rationale for this approach.
**Data presentation**
Bar graphs should only be used to present counts or proportions. If you are using bar graphs that present means/averages, it is best practice to include individual data points and/or convert the graph to a boxplot or dot-plot. You may wish to refer to this blog post about representing data distribution in plots (particularly for small datasets).
All blots/gels must be accompanied by size markers in every figure panel. In addition, please check that your blot/gel images comply with the Nature Portfolio image integrity guidelines: https://www.nature.com/nature-research/editorial-policies/image-integrity
