# Supplementary material for: High-throughput telomere length measurement at nucleotide resolution using the PacBio high fidelity sequencing platform
Source: Nat Commun. 2023 Jan 17;14:281. doi: 10.1038/s41467-023-35823-7 (PMC9845338; doi:10.1038/s41467-023-35823-7)

Supplementary information:

High-throughput telomere length measurement at nucleotide resolution using the PacBio high fidelity sequencing platform

Cheng Yong Tham[1,†], LaiFong Poon[2,†], TingDong Yan[2,3], Javier Yu Peng Koh[2], Muhammad Khairul Ramlee[2], Vania Swee Imm Teoh[1], Suihan Zhang[4], Yi Cai[2,5], Zebin Hong[8], Gina S. Lee[6], Jin Liu[7], Hai Wei Song[8], William Ying Khee Hwang [2,9,10], Bin Tean Teh[2,8,11] , Patrick Tan[1,2,12,13], Lifeng Xu[4], Angela S. Koh[6], Motomi Osato[1,14*] and Shang Li[2,15,*]

†These authors contributed equally

*Co-corresponding authors

[1]Cancer Science Institute of Singapore, National University of Singapore, 14 Medical Drive, Singapore 117599

[2]Cancer and Stem Cell Biology Program, Duke-NUS Medical School, 8 College Road, Singapore 169857

[3]School of Life Sciences, Shanghai University, 99 Shangda Road, Shanghai, China 200444

[4]Department of Microbiology and Molecular Genetics, University of California, Davis, CA USA 95616

[5]Key Laboratory of Molecular Target & Clinical Pharmacology, School of Pharmaceutical Science, Guangzhou Medical University, Guangzhou, China 511436

[6]National Heart Centre Singapore, Duke-NUS Medical School, 5 Hospital Drive, Singapore 169609

[7]Centre for Quantitative Medicine, Duke-NUS Medical School, 8 College Road, Singapore 169857

[8]Institute of Molecular and Cell Biology, Agency for Science, Technology and Research, (A*STAR), 61 Biopolis Drive, Singapore 138673

[9]Department of Haematology, Singapore General Hospital, 1 Hospital Drive, Singapore 169608

[10]Hematopoietic Stem Cell and Cellular Therapy Laboratory, Division of Medical Sciences, National Cancer Centre Singapore, 11 Hospital Drive, Singapore 169610

[11]Laboratory of Cancer Epigenome, Division of Medical Science, National Cancer Centre Singapore, 11 Hospital Drive, Singapore 169610

[12]SingHealth/Duke-NUS Institute of Precision Medicine, National Heart Centre Singapore, Singapore 168752

[13]Epigenetic and Epitranscriptomic Regulation Domain, Genome Institute of Singapore, Agency for Science, Technology and Research, 60 Biopolis Drive, Singapore 138672

[14]International Research Center for Medical Sciences, Kumamoto University, 2-2-1 Honjo, Chuo-ku, Kumamoto, Japan 860-0811

[15]Department of Physiology, Yong Loo Lin School of Medicine, National University of Singapore, 2 Medical Drive, Singapore 117597

Corresponding authors

Motomi Osato: csimo@nus.edu.sg; motomi.osato@gmail.com

Shang Li: shang.li@duke-nus.edu.sg

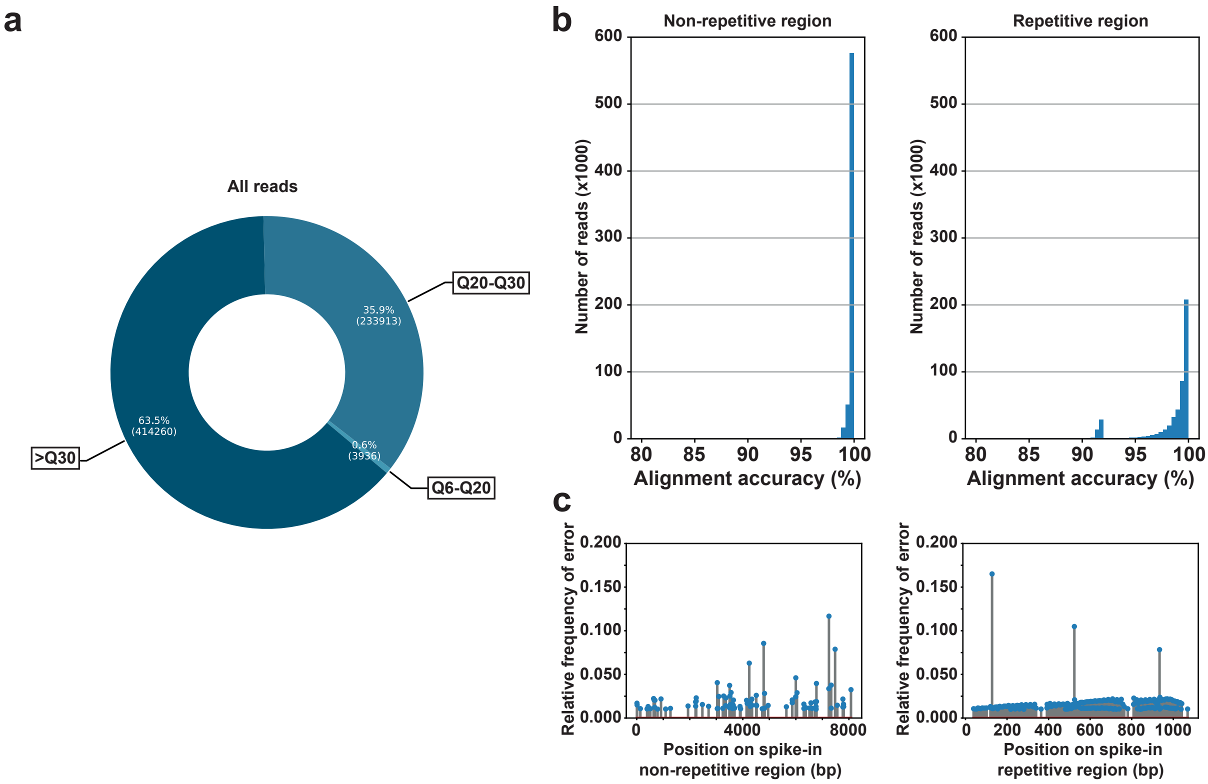

Figure S1: PacBio HiFi sequencing of DNA vector with telomeric repeats. a. Pie chart showing the accuracy of PacBio HiFi sequencing reads of the spike-in pWY82 DNA vector that contains the *Arabidopsis* telomeric sequence 5'-TTTAGGG-3'. **b.** Alignment accuracy of the non-repetitive and repetitive regions of the spike-in pWY82 DNA vector as calculated by HS-BLASTN. **c.** Relative frequency of sequencing error per base for the non-repetitive and repetitive regions of the spike-in pWY82 DNA vector. Only sequencing errors with a relative frequency greater than or equal to 0.01 are shown.

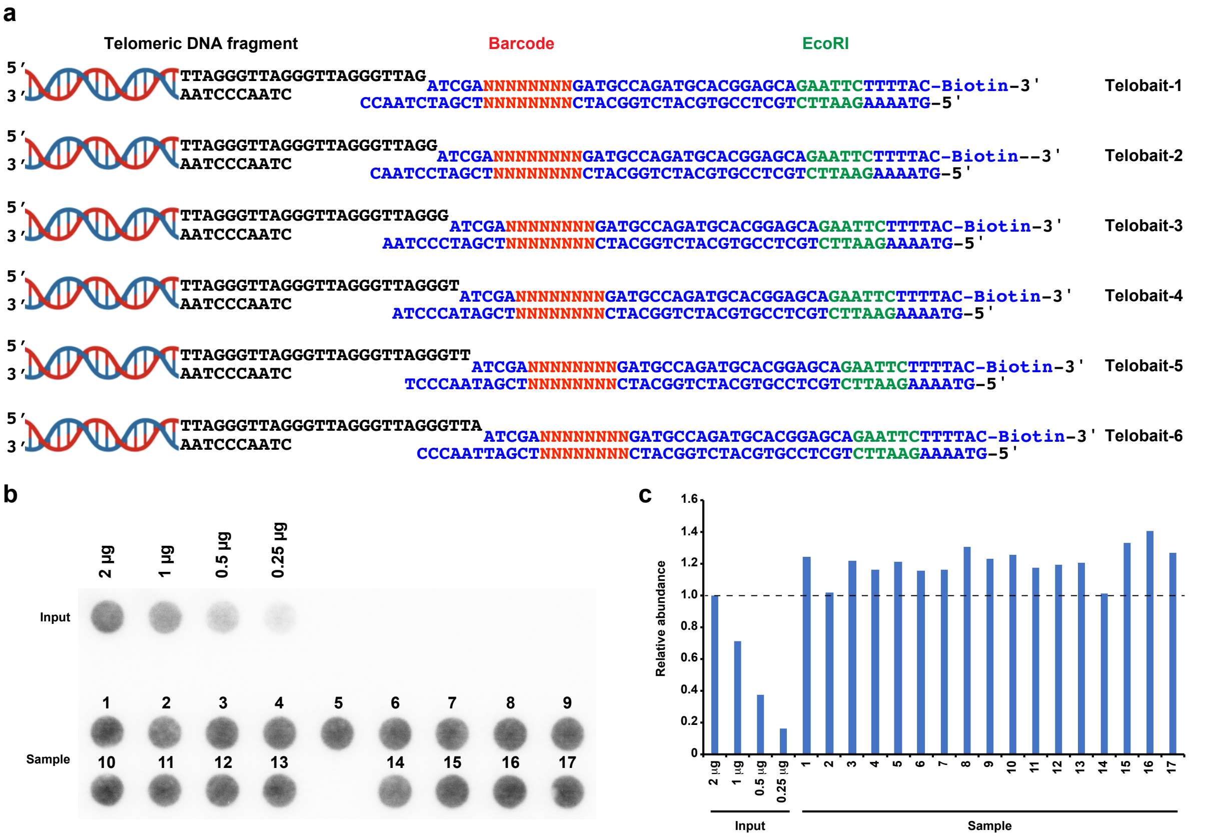

Figure S2: Telomere enrichment using telobaits. a. Schematic illustration of the design of telobaits for the enrichment of telomere-containing genomic DNA. Six telobaits were designed, each containing a single telomere repeat with all the six possible ends that can anneal to the single-stranded telomeric G-rich 3' overhangs. Each telobait also contains a unique barcode (Red), an EcoRI restriction endonuclease recognition site (green), and 3' biotin labelling. **b.** Dotblot showing the efficiency of telomere-containing genomic DNA enrichment using telobaits. The signal from a total input of 2 μg, 1 μg, 0.5 μg and 0.25 μg of genomic DNA extracted from HCT116 cells is shown on the first row. About 10 μg of genomic DNA obtained from HCT116 cells was enriched using telobaits without a barcode at a molar ratio of telobait:genomic DNA=10,000:1 (sample 1); or 12 different telobaits with unique barcodes at a molar ratio of telobait:genomic DNA=10,000:1 (sample 2 – 13); telobait without a barcode at a molar ratio telobait:genomic DNA=1,000:1 (sample 14); telobait:genomic DNA=5,000:1 (sample 15), telobait:genomic DNA=20,000:1 (sample 16); telobait:genomic DNA=50,000:1 (sample 17). **c.** Relative abundance of telomere repeat signal in **b** was quantified using ImageJ.

AATCACTTGAACCCAGAAGGCAGAGGCTGCAGTGTGCCGAGACCGTACTCCCTCTTGGTGAAAAGCTGTCTTCCAAGTTAATAACTGGGACATCAGCTCT
CCGCAGTAATAGCTCCTTTTCAGTTTTAATTTGCAGAACTGATGGGGATTAATAACTGGCGCTCCGACTTTAAGTGGTGCAGGAGGCGGCCAGTAGGGGA
CGCCAGCCGTTACACCGGGAGCAAGAGGGCCGTCCGTAGTCCCCATCTGCCTGCATGTGGCGTGCAGCGACGACAATGGCAGCAAGAGGGCCCGGCAGTG
TGCCCAGCTGCCAGCAGGCGGGTGTGCTGCCACTATAATGTGAGGAAGAGGGCCCTGCAATGTCCCTAGCTGCTAGCAGGCGGCGTGCCACCACTATACT
GCGAGCAAGAGAGCCCTGCCGTGCCCCTGCGCCAGCACGGGGCGCTGGACACCACTGTAAGCAAGAGGGCCCTGCAGTTGTCCTAGTCGCCAGTAGGGGG
CGCAATGGCAGAGCACCGTGGGCAAGCTGGTCCTGTAGTGCCCGGCTGCAAGCAGGGGGCGCCCGAAACGGGCTTTTCAGATTACTCAGGTTCAACTCGT
CTCTGCGCCGCGCCGCCGGGGACGTGTGTCTCTGCGCGTGCACCGCACCACCTCCGCGCTCCCCGCCGGGCGGCGCGCGACTGTGCGACTGCAACACTCC
CAGCCACCCTCAGCCCAGCGACGTGCGTCTCTGCGCCTGCGCCGCGCCTCACTCCCGCCCGCCCAGCGACCCCTCCCCTCCGGGGAGGCGCCGGCGTGCG
TCTACGCCCTGCGCCGCGTCTCCCCAACAGCGGCGCGCCTCTCTGCGCCTGCGCCGGCGCGCCGCCCCCTCTCTGCGCCTGCGCCGGCGCGCCGCCCCCT
CTCTGCGCCTGCGCCGGCGCGCCGACCCCCTCTCTGCGCCTGCGCCGGCGCGCCGACCCCTCTCTGCGCCTGCGCCGGCGCGCCGACCCCCTCTCTGCGC
CTGCGCCGGCGCGCCGACCCCCCTCTTGCGCCTGCGCCGGCGCGCCGACCCCCTCTCTGCGCCTGCGCCGGCGCGCCGACCCCCTCTCTGCGCCTCCGCT
GGCGCGCCGCCTTTGCGAGGGCGGAGTTGCGTTCTCTTTAGCACACACCCGGAGAGCATCGCGACGGCGGAGCTGCGTTCTCCTCTGCACAGACTTCGGG
GCTATTGCGAAGGCGGAGCACAGTTCTTCTCAGGTCAGACCCGGGTGGGCGGGCTGAGGGCACTGCGAGGGCGGAGCTGCGTTCTGTTCAGCACAGACCT
GGGGGGTTAGGGTTAGGGGTGCGGTTGGGGTTGGGGTTGGGGTTAGGGTTAGGGTTAGGGTTAGGGTTAGGGTTAGGGGTTAGGGGTTAGGGTTGGGGTT
GGGGTTGGGGTTGGGGTTCGGGTTAAGGGTTAGGGTTAGGGTTAGGGCTAGGGCTAGGGCTAGGGCTAGGGCTAGGGTTAAGGGTTAGGGTTGGGGTTGG
GGTTGGGGTTGGGGTTGGGGTTAGGGTTAGGGTTAGGGTTAGGGTTAGGGTAGGGTTAGGGTTAGGGTTAGGGTTGGGTTAGGGTTAGGGTTAGGGTTAG
GGTTAGGGTAGGGTTAGGGTGAGGGTGAGGGTGAGGGTGAGGGTGAGGGTGAGGGTGAGGTTAGGGTTAGGGTAGGGTTAGGGTTAGGGTTAGGGTTAGG
GTTAGGTTAGGGTTAGGGTTAGGGGTTAGGGTTAGGGGTTGGGGTTGGGGTTGGGGTTGGGGTTAGGGTTAGGGTTAGGGTTAGGGTAAGGGTTAAGGGT
TAGGGTTAGGGTTAGGGTTAGGGTTAGGGTTAGGGTTAGGGTTGGGTTAGGGTTAGGGTTAGGGTTTAGGGTTAGGGTTAGGGTTAGGGTTAGGGTTCGG
GTTCGGGTTAGGGTTCGGGTTCGGGTTCGGGTTCGGGTTAGGGTTAGGGTTAGGGTTCGGGTTAGGGTTCGGGTTCGGGTTCGGGTTAGGGTTAGGGTTA
GGGTTCGGGTTAGGGTTCGGGTTCGGGTTCGGGTTCGGGTTCGGGTTCGGGTTAGGGGTTCGGGTTAGGGGTTCGGGTTCGGGTTAGGGGTTAGGGGTTA
GGGTTAGGGTTAGGGTTAGGGTTAGGGTTAGGGTTAGGGGTTAGGGTTAGGGTTAGGGTTAGGGTTAGGGTTAGGGGTTAGGGGTTAGGGTTAGGGTTAG
GGTTAGGGTTAGGGTTAGGGGTTAGGGTTAGGGTTAGGGTTAGGGTTAGGGTTAGGGTTAGGGTAGGGTTAGGGTAGGGTTAGGGTTAGGGTTAGGGTTA
GGGTTAGGTTTGGGTTAGGGTTAGGGTTAGGGTTAGGGTTAGGGTTAGGGTTAGGGTTAGGGTTTTAGGGTTTGGGTTAGGGTTAGGGTTAGGGTTAGGG
TTAGGGGTTAGGGTTAGGGTTAGGGTTAGGGTTAGGGTTAGGGTTAGGTTAGGGTTAGGGTTAGGGTTAGGGTTAGGGTAGGGTTAGGGTTAGG
GTTAGGGTTAGGGTTAGGGTTAGGGTTAGGGTTAGGGTTAGGGTTAGGGGTTAGGGTTAGGGTTAGGGTTAGGGTTAGGGTTAGGGTTAGGGTTAGGGTT
AGGGTTAGGGTTAGGGTTAGGGTTAGGGTTAGGGTTAGGGTTAGGGTTAGGGTTAGGGTTAGGGTTAGGGGTTAGGGTTAGGGTTAGGGTTAGGGTTAGG
GGTTAGGGTTAGGGTTAGGGTTAGGGTTAGGGTTAGGGTTAGGGTTAGGGTTAGGGTTAGGGTTAGGGTTAGGGGTTAGGGGTTAGGGTTAGGG
TTAGGGTTAGGGTTAGGGTTAGGGTTAGGGGTTGGGGTTGGGGTTGGGGTTAGGGTTAGGGTTAGGGTTAGGGTTAGGGTTAGGGGTTAGGGTTGGGGTT
GGGGTTGGGGTTGGGGTTGGGGTTGGGGTTGGGGTTAGGGTTAGGGTTAGGGTTTAGGGTTTAGGGTTAGGGTTTAGGGTTAGGGTTAGGGTTAGGGTTA
GGGTTAGGGTTAGGGTTAGGGTTAGGGTTAGGGTTAGGGTTAGGGTTAGGGTTAGGGTTAGGGTTAGGGTTAGGGTTAGGGTTAGGGTTAGGGT
TAGGGTTAGGGTTAGGGTTAGGGTTAGGGTTAGGGTTAGGGTTAGGGTTAGGGTTAGGGTTAGGGTTAGGGTTAGGGTTAGGGTTAGGGTTAGG
TTAGGGTTAGGGTTAGGGTTAGGGTTAGGGTTAGGGTTAGGGTTAGGGTTAGGGTTAGGGTTAGGGTTAGGGTTAGGGTTAGGGTTAGGGTTAGGGTTAG
GGTTAGGGTTAGGGTTAGGGTTAGGGTTAGGGTTAGGGTTAGGGTTAGGTTAGGGTTAGGGTTAGGGTTAGGGTTAGGGTTAGGGTTAGGGTTAGGGTTA
GGGTTAGGGTTAGGGTTAGGGTTAGGGTTAGGGTTAGGGTTAGGGTTAGGGTTAGGGTTAGGGTTAGGGTTAGGGTTAGGGTTAGGGTTAGGGT
TAGGGTTAGGGTTAGGGTTAGGGTTAGGGTTAGGGTTAGGGTTAGGGTTAGGGTTAGGGTTAGGGTTAGGGTTAGGGTTAGGGTTAGGGTTAGG
GTTAGGTTAGGGTTAGGGTTAGGGTTAGGGTTAGGGTTAGGGTTAGGGTTAGGTTAGGGTTAGGGTTAGGGTTAGGGTTAGGGTTAGGGTTAGGGTTAGG
GTTAGGGTTAGGGTTAGGGTTAGGTTAGGGTTAGGGTTAGGGTTAGGGTTAGGGTTAGGGTTAGGGTTAGGGTTAGGGTTAGGGTTAGGGTTAG
GGTTAGGGTTAGGGTTAGGGTTAGGGTTAGGGTTAGGGTTAGGGTTAGGGTTAGGGTTAGGGTTAGGGTTAGGGTTAGGGTTAGGGTTAGGGTT
AGGGTTAGGGTTAGGGTTAGGGTTAGGGTTAGGGTTAGGGTTAGGGTTAGGGTTAGGGTTAGGGTTAGGGTTAGGGTTAGGGTTAGGGTTAGGG
TTAGGGTTAGGGTTAGGGTTAGGGTTAGGGTTAGGGTTAGGGTTAGGGTTAGGGTTAGGGTTAGGGTTAGGGTTAGGGTTAGGGTTAGGGTTAGGGTTAG
GGTTAGGGTTAGGTTAGGGTTAGGGTTAGGGTTAGGGTTAGGGTTAGGGTTAGGGTTAGGGTTAGGGTTAGGGTTAGGGTTAGGGTTAGGGTTA
GGGTTAGGGTTAGGGTTAGGGTTAGGGTTAGGGTTAGGGTTAGGGTTAGGGTTAGGGTTAGGGTTAGGGTTAGGGTTAGGGTTAGGGTTAGGGT
TAGGGTTAGGGGTTAGGGTTAGGGTTAGGGTTAGGGTTAGGGTTAGGGTTAGGGTTAGGGTTAGGGTTAGGGTTAGGGTTAGGGTTAGGGTTAG
GGTTAGGGTTAGGGTTAGGGTTAGGGTTAGGGTTAGGGTTAGGGTTAGGGTTAGGTTAGGGTTAGGGTTAGGGTTAGGGTTAGGGTTAGGGTTA
GGGTTAGGGTTAGGGTTAGGGTTAGGGTTAGGGTTAGGGTTAGGGTTAGGGTTAGGGTTAGGGTTAGGGTTAGGGTTAGGGTTAGGGTTAGGGT
TAGGTTAGGGTTAGGGTTAGGGTTAGGGTTAGGGTTAGGGTTAGGGTTAGGGTTAGGTTAGGGTTAGGGTTAGGGTTAGGGTTAGGGTTAGGGT
TAGGGTTAGGGTTAGGGTTAGGGTTAGGGTTAGGGTTAGGGTTAGGGTTAGGGTTAGGGTTAGGGTTAGGGTTAGGGTTAGGGTTAGGGTTAGG
GTTAGGGTTAGATCGACGGTTCAAGATGCCAGATGCACGGAGCAGAATT

Figure S3: The sequence of a typical telomere-containing PacBio HiFi sequencing read. The canonical telomere (5'-TTAGGG-3')n sequence is highlighted in red. The MnlI and HphI restriction endonuclease recognition sites inside TVSs are highlighted as brown and blue rectangles, respectively. The telobait sequence and the barcode within are highlighted in green and blue, respectively.

a

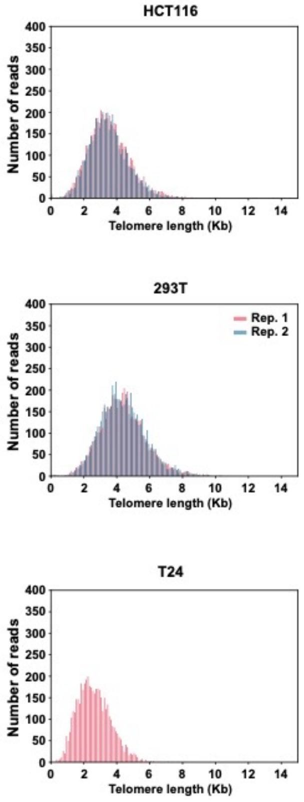

b

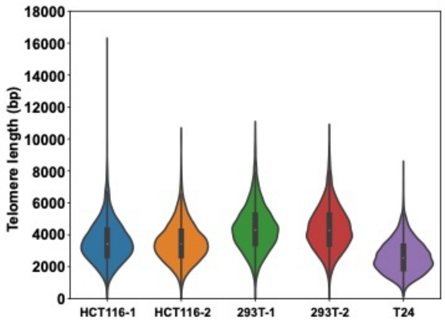

c

	HCT116-1	HCT116-2	293T-1	293T-2	T24
No. of reads	5615	5287	6038	6303	4752
Mean (bp)	3545	3519	4399	4393	2637
Median (bp)	3426	3414	4314	4284	2544
IQR (bp)	2688-4290	2694-4224	3444-5232	3420-5244	1884-3288
Min (bp)	12	12	12	12	138
Max (bp)	16308	10692	11088	10914	8604

Figure S4: Telomere length measurement in culture cells using PacBio HiFi sequencing. a. and b. Histograms and violin plots of telomere length distribution in HCT116 (two biological replicates), 293T (two biological replicates) and T24 cells derived from an independent PacBio HiFi sequencing run. **c.** Table showing the characteristics of telomere length distribution in HCT116, 293T, and T24 cells as shown in violin plots in **b**. Within each violin, the white center circle denotes the median value, the bounds of box represent the 25th to 75th percentile values, the whiskers represent adjacent values within 1.5 interquartile range, the ends of the whiskers depict the minimum and maximum values within the range, and the violin shape reflect the kernel density plot of the entire dataset.

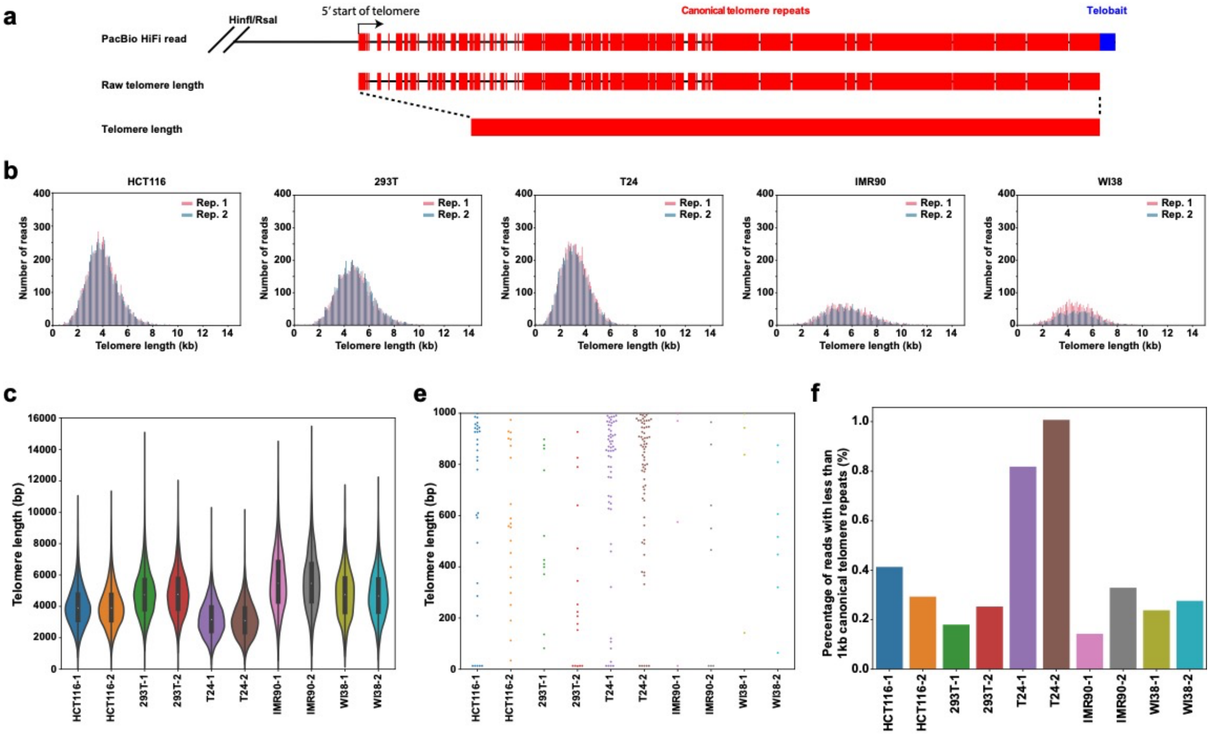

d

	HCT116-1	HCT116-2	293T-1	293T-2	T24-1	T24-2	IMR90-1	IMR90-2	WI38-1	WI38-2
No. of reads	7256	7162	6122	6322	6968	6553	2788	2432	1681	2535
Mean (bp)	4005	3997	4830	4852	3223	3163	5614	5599	4785	4733
Median (bp)	3908	3887	4734	4756	3147	3083	5472	5464	4739	4660
IQR (bp)	3140-4742	3127-4729	3824-5673	3855-5743	2437-3927	2364-3864	4317-6820	4332-6694	3646-5774	3669-5714
Min (bp)	13	34	82	12	13	13	13	13	142	64
Max (bp)	11051	11341	15084	12027	10290	10158	14515	15474	11739	12236

Figure S5: Raw telomere length measurement in culture cells using PacBio HiFi sequencing data as presented in Figure 2. a. Schematic illustration for the estimation of raw telomere length or telomere length with or without the inclusion of heterogenous TVSs in sequencing reads, respectively. **b. and c.** Histograms and violin plots of raw telomere length distribution in HCT116, 293T, T24, IMR90 (PD47) and WI38 (PD40) cells obtained from two biological replicates (Rep.) in a single sequencing run. PD: population doubling. Within each violin, the white center circle denotes the median value, the bounds of box represent the 25th to 75th percentile values, the whiskers represent adjacent values within 1.5 interquartile range, the ends of the whiskers depict the minimum and maximum values within the range, and the violin shape reflect the kernel density plot of the entire dataset. **d.** Table showing the characteristics of raw telomere length distribution in HCT116, 293T, T24, IMR90 (PD47) and WI38 (PD40) cells obtained from two biological replicates as shown in the violin plot in **c**. **e.** Beeswarm plot showing the over-representation of extremely short telomeres (<1000 bp raw telomere length) in sequencing reads, specifically from T24 cells. **f.** Bar graph showing the over-representation of extremely short telomeres (<1000 bp raw telomere length) in sequencing reads, specifically in T24 cells after normalization.

a

	Run 1-1	Run 1-2	Run 2-1	Run 2-2
**HCT116**	**3539 bp**	**3509 bp**	**3512 bp**	**3498 bp**
**293T**	**4395 bp**	**4389 bp**	**4286 bp**	**4299 bp**
**T24**	**2634 bp**		**2737 bp**	**2672 bp**

b

HCT116 (mean: 3519.0, SD: 1231.8, Total reads: 25,320)

Sample size	1,000	2,000	5,000	10,000	15,000	20,000
**Mean of means**	3519.1	3518.6	3519.0	3519.1	3518.8	3518.9
**SE of mean**	39.1	27.4	17.5	12.2	9.9	8.7
**1.96*SE of mean**	76.6	53.7	34.3	23.9	19.4	17.1
**95% CI**	3442.4 - 3595.6	3465.3 - 3572.7	3484.7 - 3553.3	3495.1 - 3542.9	3499.6 - 3538.4	3501.9 - 3536.1

c

293T (mean: 4347.0, SD: 1404.5, Total reads: 24,785)

Sample size	1,000	2,000	5,000	10,000	15,000	20,000
**Mean of means**	4346.9	4347.1	4347.3	4347.0	4347.1	4347.1
**SE of mean**	44.6	31.4	19.8	14.1	11.4	9.9
**1.96*SE of mean**	87.4	61.5	38.8	27.6	22.3	19.4
**95% CI**	4259.6 - 4434.4	4285.5 - 4408.5	4308.2 - 4385.8	4319.4 - 4374.6	4324.7 - 4369.3	4327.6 - 4366.4

Figure S6: Accuracy and consistency of telomere length measurement using PacBio HiFi sequencing. a. Estimated mean telomere length in biological replicates of HCT116, 293T and T24 cells derived from two independent PacBio HiFi sequencing runs. **b.** and **c.** The predicted standard error (SE) and 95% confidence interval (CI) of mean calculated by random sampling at different sequencing depths using the pooled telomere-containing reads from HCT116 and 293T cells in two independent sequencing runs, respectively. The bootstrap method was used where telomeric reads were resampled from the actual sample with replacement for 10,000 iterations for the different sample sizes.

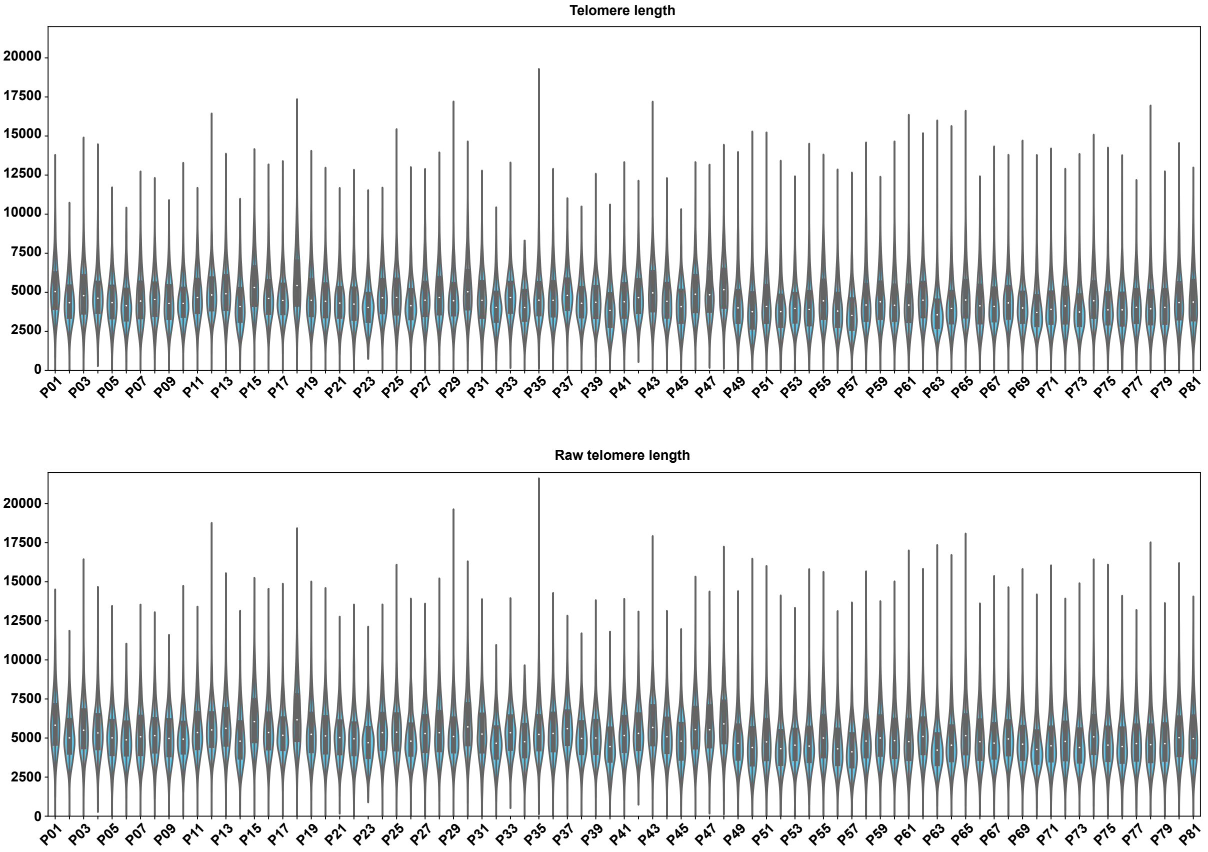

Figure S7: Violin plots showing telomere length and raw telomere length distribution in 81 patient PBL samples from Singapore cohort. Please refer to the Source Data 2 file for the n value of each sample. Within each violin, the white center circle denotes the median value, the bounds of box represent the 25th to 75th percentile values, the whiskers represent adjacent values within 1.5 interquartile range, the ends of the whiskers depict the minimum and maximum values within the range, and the violin shape reflect the kernel density plot of the entire dataset.

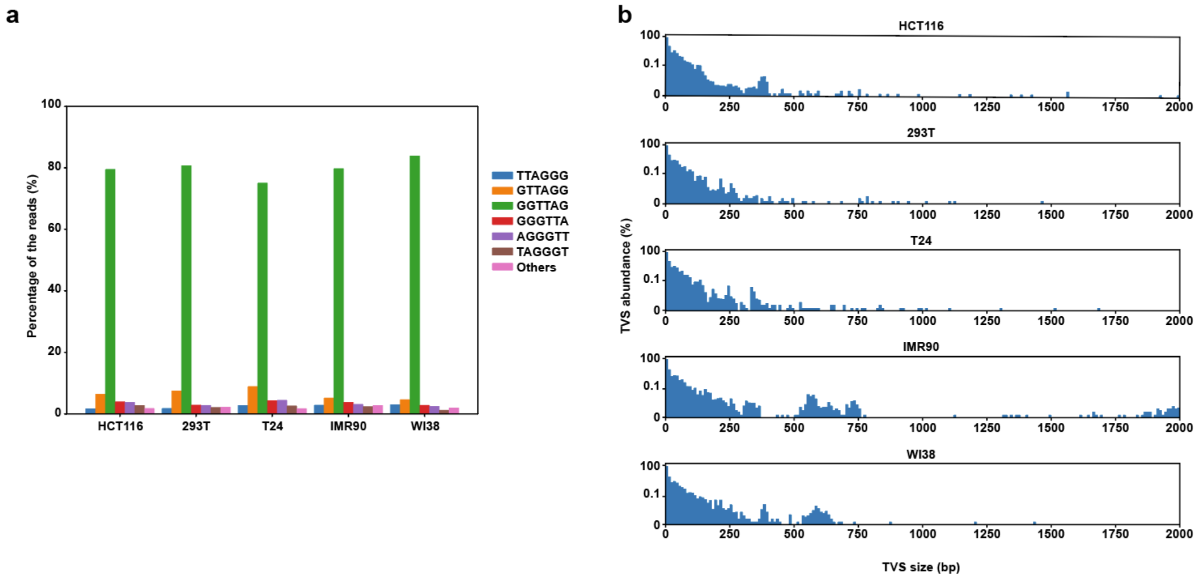

Figure S8: Proportion of different G strand end sequences and TVS size abundance distributions in culture cell lines. a. Bar graph showing the percentage of different G strand end sequences across culture cell lines. Like patient PBL samples, the most common G strand end observed is 5'-GGTTAG-3'. **b.** Histograms showing the unique TVS size abundance profile of each culture cell line. The Y-axes are presented in logarithmic scale. Only TVSs with sizes less than or equal to 2kb are shown.

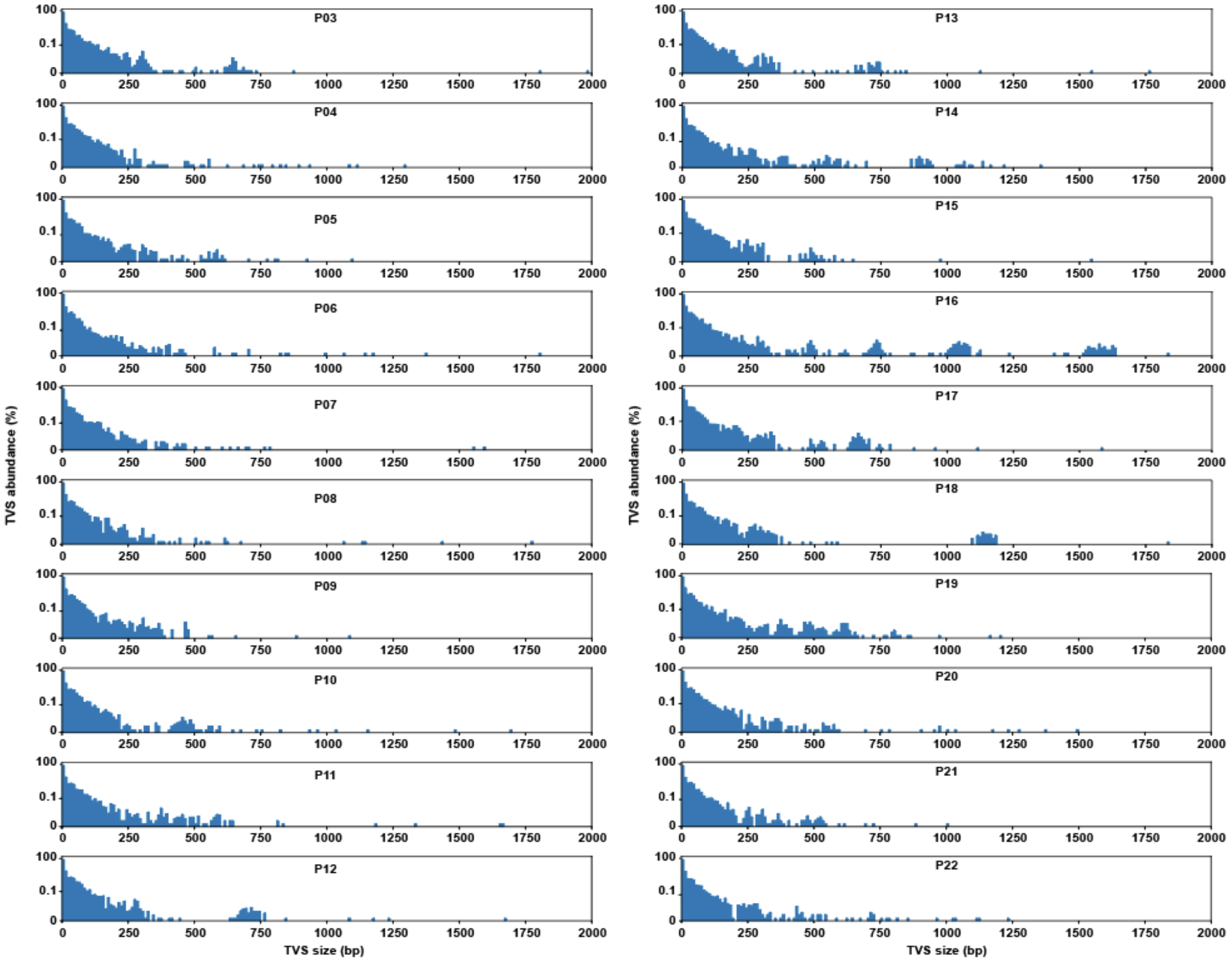
TVS abundance (%)
TVS size (bp)
P03
P04
P05
P06
P07
P08
P09
P10
P11
P12
P13
P14
P15
P16
P17
P18
P19
P20
P21
P22
100
0.1
0
0
250
500
750
1000
1250
1500
1750
2000

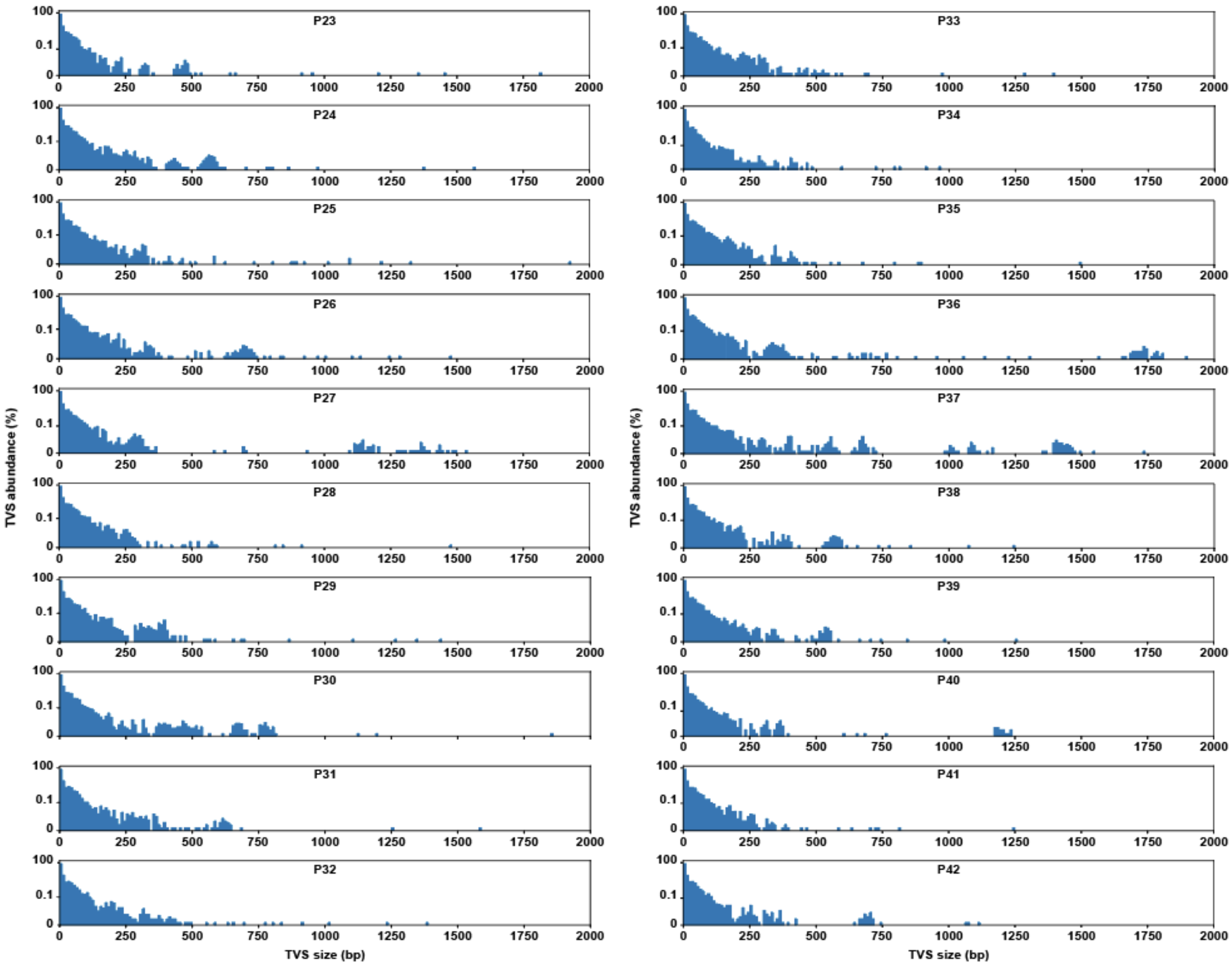
P23
P24
P25
P26
P27
P28
P29
P30
P31
P32
P33
P34
P35
P36
P37
P38
P39
P40
P41
P42
100
0.1
0
0
250
500
750
1000
1250
1500
1750
2000
TVS abundance (%)
TVS size (bp)

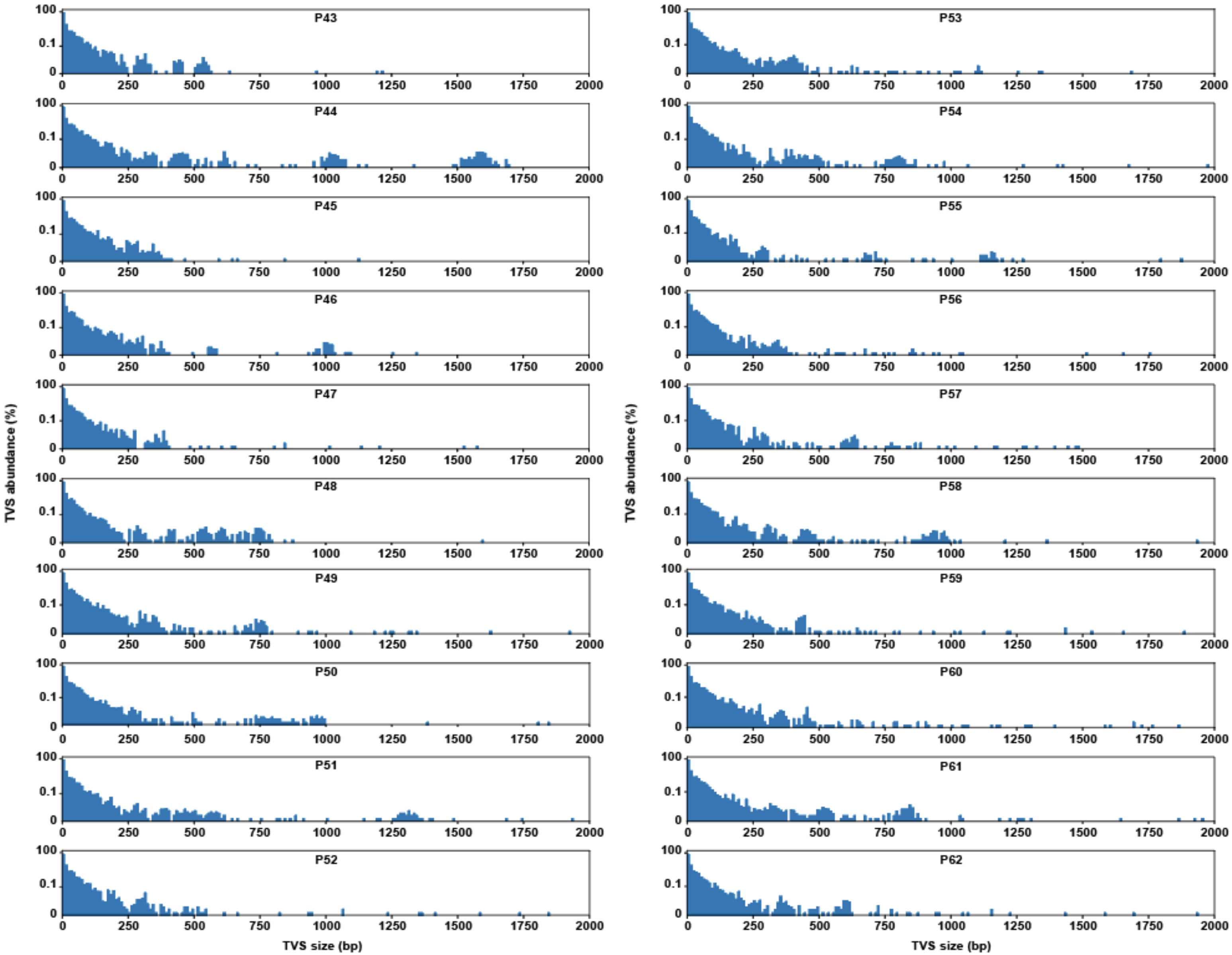

TVS abundance (%)
TVS size (bp)
P43
P44
P45
P46
P47
P48
P49
P50
P51
P52
P53
P54
P55
P56
P57
P58
P59
P60
P61
P62
100
0.1
0
0
250
500
750
1000
1250
1500
1750
2000

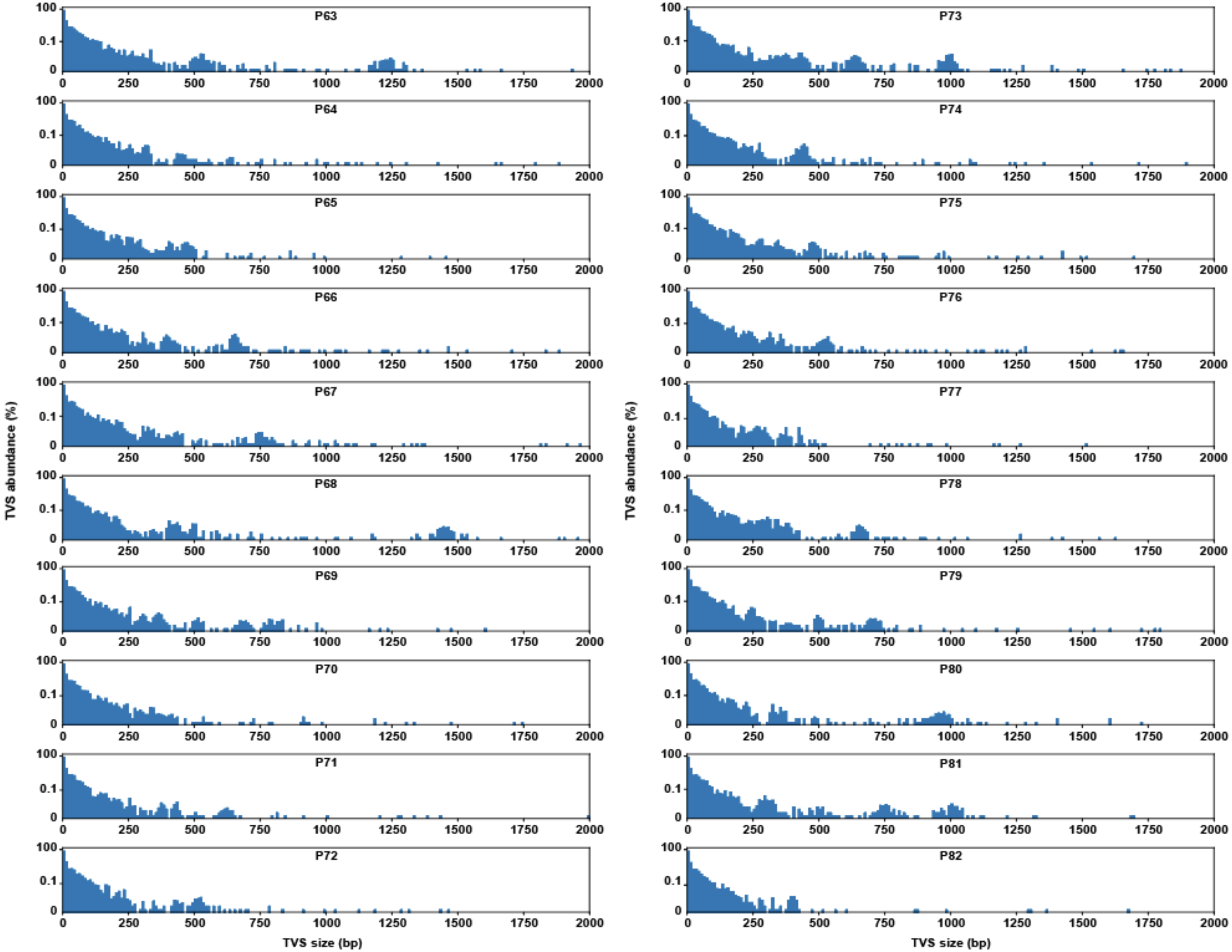

TVS abundance (%)
TVS size (bp)
P63
P64
P65
P66
P67
P68
P69
P70
P71
P72
P73
P74
P75
P76
P77
P78
P79
P80
P81
P82
100
0.1
0
0
250
500
750
1000
1250
1500
1750
2000

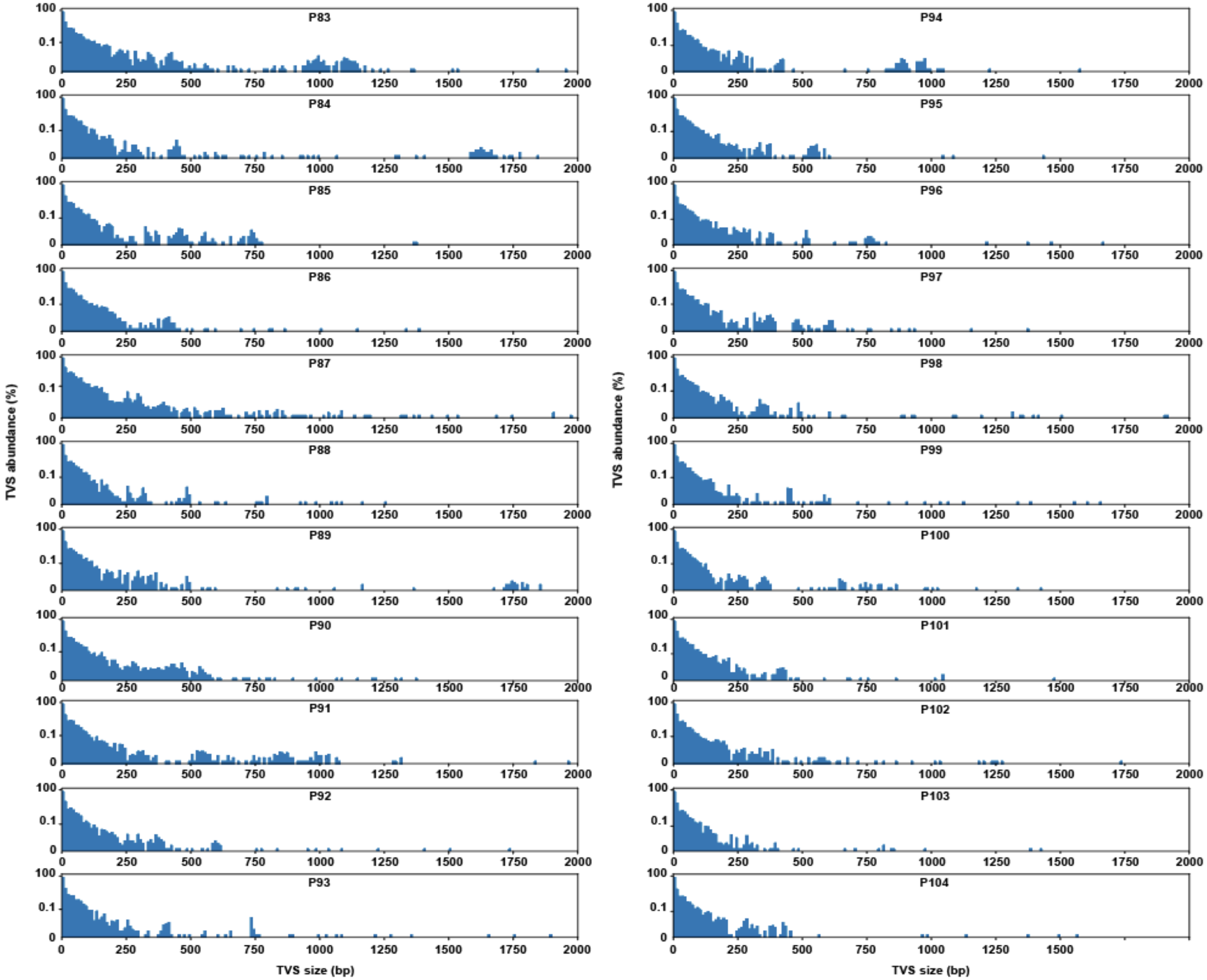
TVS abundance (%)
TVS abundance (%)
P83
P84
P85
P86
P87
P88
P89
P90
P91
P92
P93
P94
P95
P96
P97
P98
P99
P100
P101
P102
P103
P104
100
0.1
0
0
250
500
750
1000
1250
1500
1750
2000
TVS size (bp)
TVS size (bp)

Figure S9: The unique distribution of TVS size abundance profile in patient samples (P03-P104).

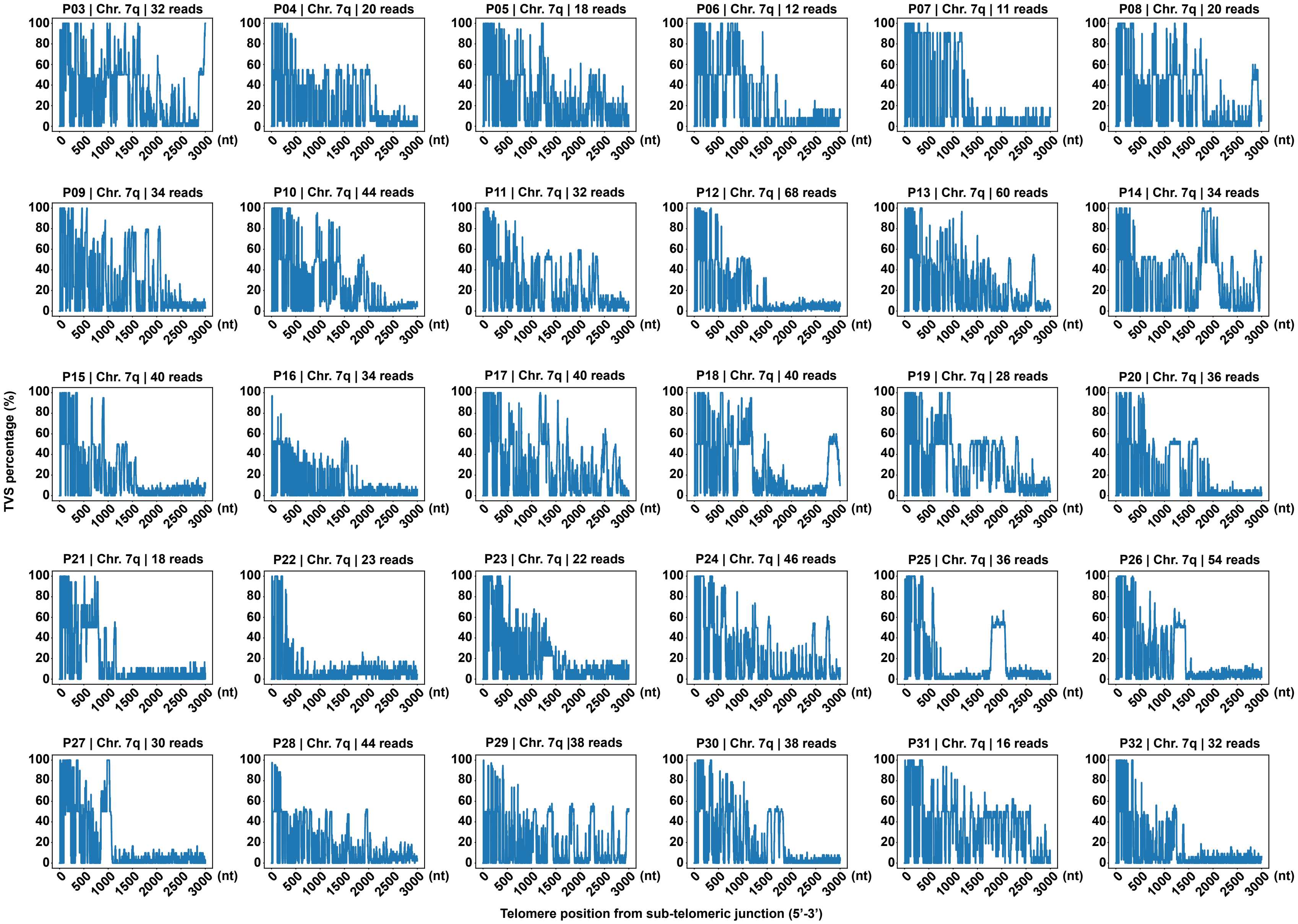
P03 | Chr. 7q | 32 reads
P04 | Chr. 7q | 20 reads
P05 | Chr. 7q | 18 reads
P06 | Chr. 7q | 12 reads
P07 | Chr. 7q | 11 reads
P08 | Chr. 7q | 20 reads
P09 | Chr. 7q | 34 reads
P10 | Chr. 7q | 44 reads
P11 | Chr. 7q | 32 reads
P12 | Chr. 7q | 68 reads
P13 | Chr. 7q | 60 reads
P14 | Chr. 7q | 34 reads
P15 | Chr. 7q | 40 reads
P16 | Chr. 7q | 34 reads
P17 | Chr. 7q | 40 reads
P18 | Chr. 7q | 40 reads
P19 | Chr. 7q | 28 reads
P20 | Chr. 7q | 36 reads
P21 | Chr. 7q | 18 reads
P22 | Chr. 7q | 23 reads
P23 | Chr. 7q | 22 reads
P24 | Chr. 7q | 46 reads
P25 | Chr. 7q | 36 reads
P26 | Chr. 7q | 54 reads
P27 | Chr. 7q | 30 reads
P28 | Chr. 7q | 44 reads
P29 | Chr. 7q |38 reads
P30 | Chr. 7q | 38 reads
P31 | Chr. 7q | 16 reads
P32 | Chr. 7q | 32 reads
TVS percentage (%)
(nt)
Telomere position from sub-telomeric junction (5'-3')

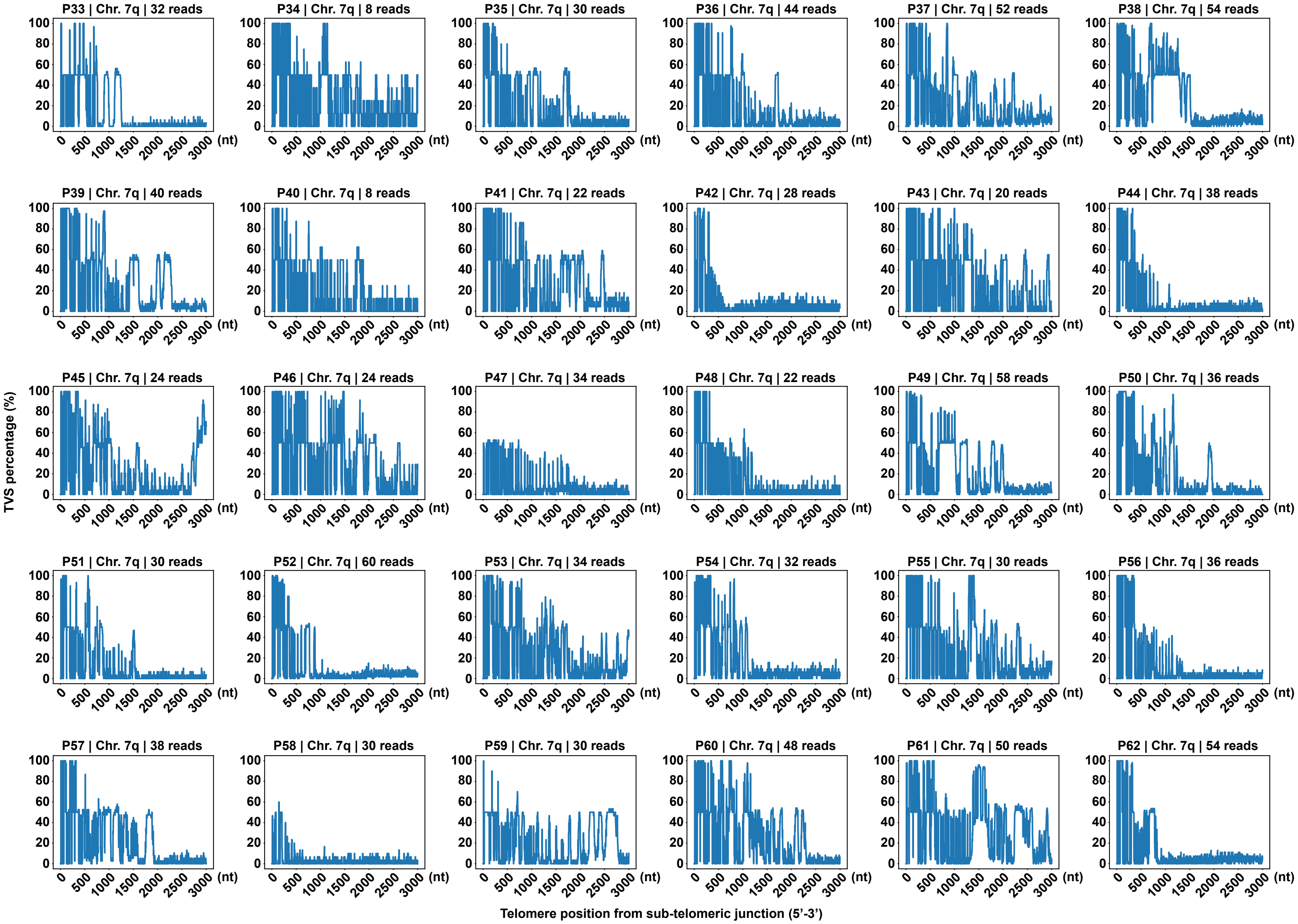
P33 | Chr. 7q | 32 reads
P34 | Chr. 7q | 8 reads
P35 | Chr. 7q | 30 reads
P36 | Chr. 7q | 44 reads
P37 | Chr. 7q | 52 reads
P38 | Chr. 7q | 54 reads
P39 | Chr. 7q | 40 reads
P40 | Chr. 7q | 8 reads
P41 | Chr. 7q | 22 reads
P42 | Chr. 7q | 28 reads
P43 | Chr. 7q | 20 reads
P44 | Chr. 7q | 38 reads
P45 | Chr. 7q | 24 reads
P46 | Chr. 7q | 24 reads
P47 | Chr. 7q | 34 reads
P48 | Chr. 7q | 22 reads
P49 | Chr. 7q | 58 reads
P50 | Chr. 7q | 36 reads
P51 | Chr. 7q | 30 reads
P52 | Chr. 7q | 60 reads
P53 | Chr. 7q | 34 reads
P54 | Chr. 7q | 32 reads
P55 | Chr. 7q | 30 reads
P56 | Chr. 7q | 36 reads
P57 | Chr. 7q | 38 reads
P58 | Chr. 7q | 30 reads
P59 | Chr. 7q | 30 reads
P60 | Chr. 7q | 48 reads
P61 | Chr. 7q | 50 reads
P62 | Chr. 7q | 54 reads
TVS percentage (%)
0
20
40
60
80
100
0
500
1000
1500
2000
2500
3000
(nt)
Telomere position from sub-telomeric junction (5’-3’)

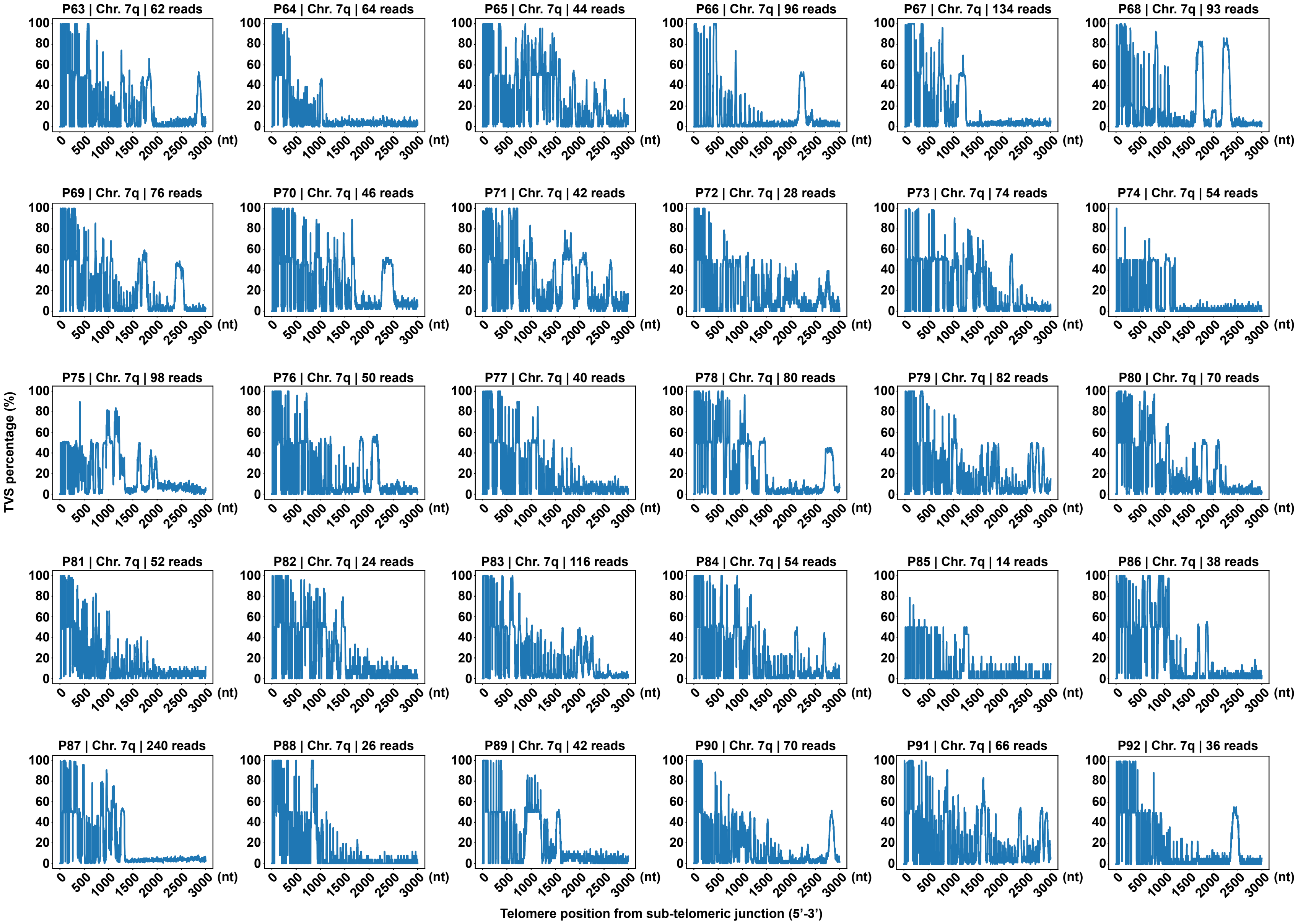
P63 | Chr. 7q | 62 reads
P64 | Chr. 7q | 64 reads
P65 | Chr. 7q | 44 reads
P66 | Chr. 7q | 96 reads
P67 | Chr. 7q | 134 reads
P68 | Chr. 7q | 93 reads
P69 | Chr. 7q | 76 reads
P70 | Chr. 7q | 46 reads
P71 | Chr. 7q | 42 reads
P72 | Chr. 7q | 28 reads
P73 | Chr. 7q | 74 reads
P74 | Chr. 7q | 54 reads
P75 | Chr. 7q | 98 reads
P76 | Chr. 7q | 50 reads
P77 | Chr. 7q | 40 reads
P78 | Chr. 7q | 80 reads
P79 | Chr. 7q | 82 reads
P80 | Chr. 7q | 70 reads
P81 | Chr. 7q | 52 reads
P82 | Chr. 7q | 24 reads
P83 | Chr. 7q | 116 reads
P84 | Chr. 7q | 54 reads
P85 | Chr. 7q | 14 reads
P86 | Chr. 7q | 38 reads
P87 | Chr. 7q | 240 reads
P88 | Chr. 7q | 26 reads
P89 | Chr. 7q | 42 reads
P90 | Chr. 7q | 70 reads
P91 | Chr. 7q | 66 reads
P92 | Chr. 7q | 36 reads
TVS percentage (%)
Telomere position from sub-telomeric junction (5'-3')

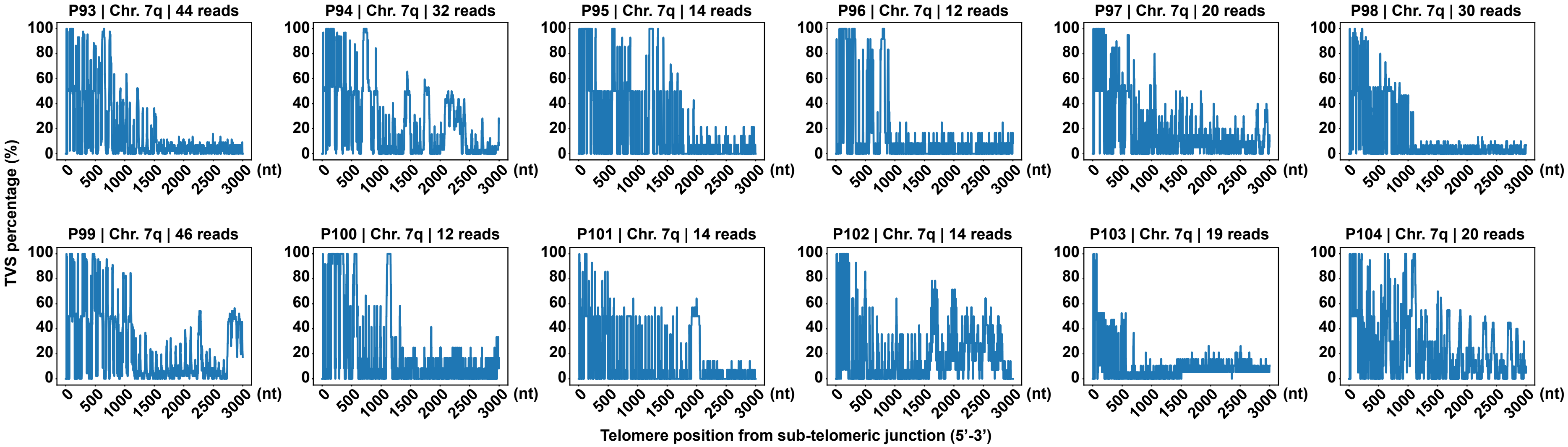

Figure S10: TVS signatures of individual patient samples (P03 to P104) for Chr. 7q. Longer and more abundant TVSs are presented near the sub-telomeric ends of the telomere repeat regions in the patient samples as shown by high TVS percentages.

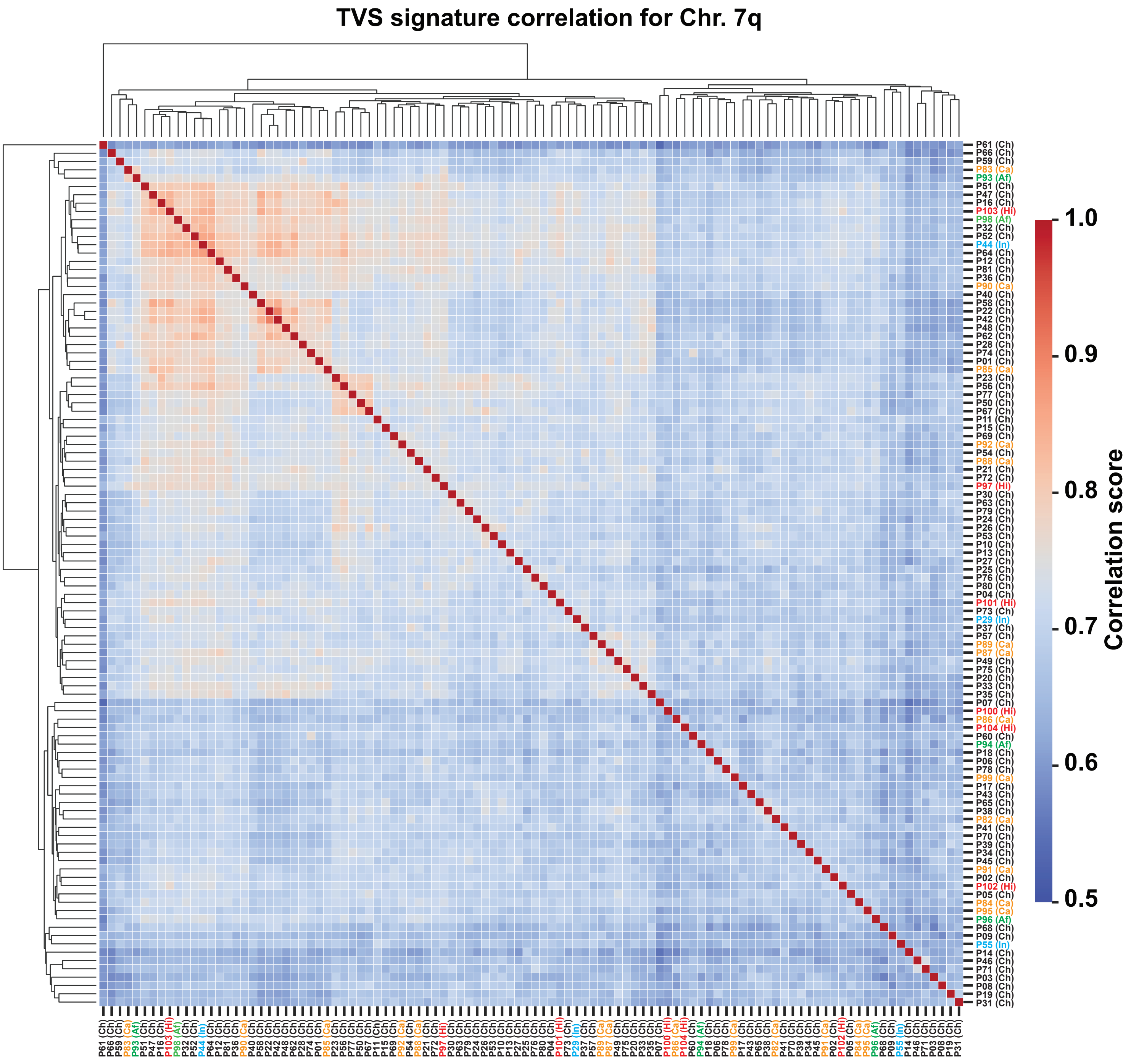

Figure S11: The dendrogram of TVS signature correlation for Chr. 7q from patient samples (P01 to P104). The TVS signatures were pairwise compared by Euclidean distance and were found to be unique to each patient. The ethnicity of each patient sample is color-coded. Af=African American; Ch=Chinese; Ca=Caucasian; Hi=Hispanic, In=Indian.

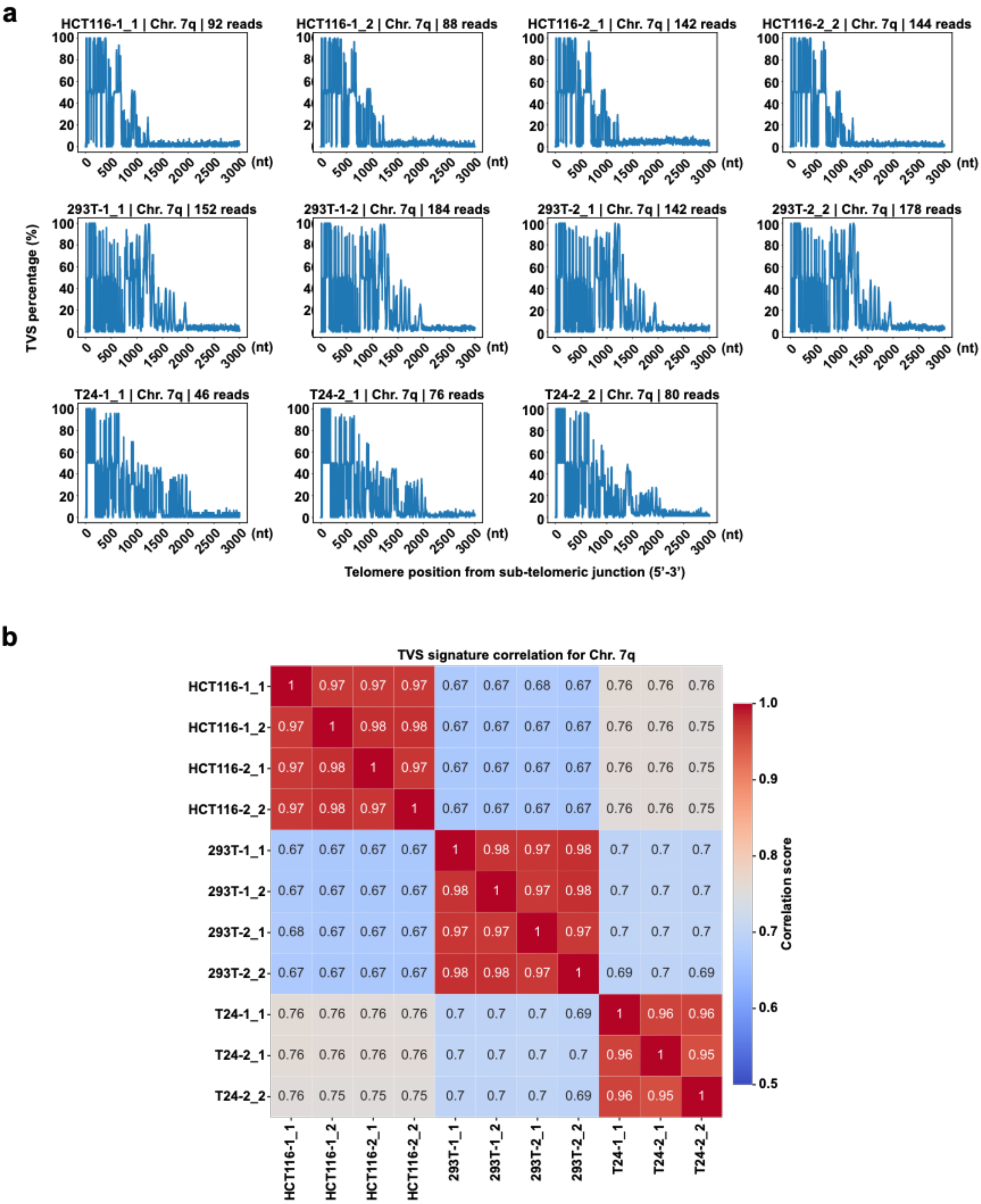

Figure S12: The consistency of TVS signature correlation of Chr. 7q from biological replicates of cell line samples. a. TVS signature plots of biological replicates of cell line samples from independent sequencing runs. **b.** The TVS signature correlation of Chr. 7q from biological replicates of cell line samples. Source data are provided in Source Data 2.

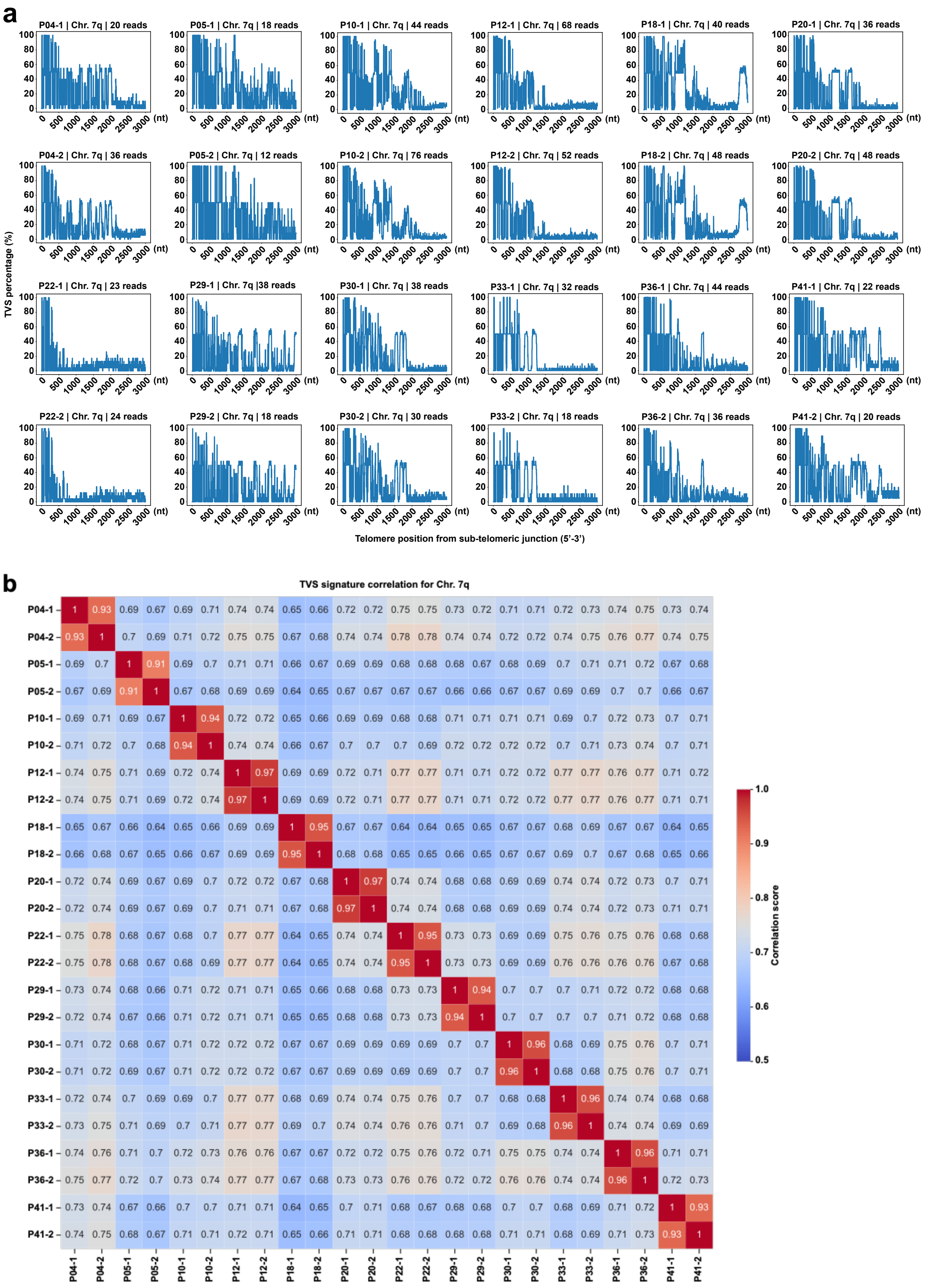

Figure S13: The TVS signature correlation of Chr. 7q from biological replicates of patient samples. A. TVS signature plots of biological replicates of patient samples from independent sequencing runs. **B.** The TVS signature correlation of Chr. 7q from biological replicates of patient samples. Source data are provided in Source Data 2.

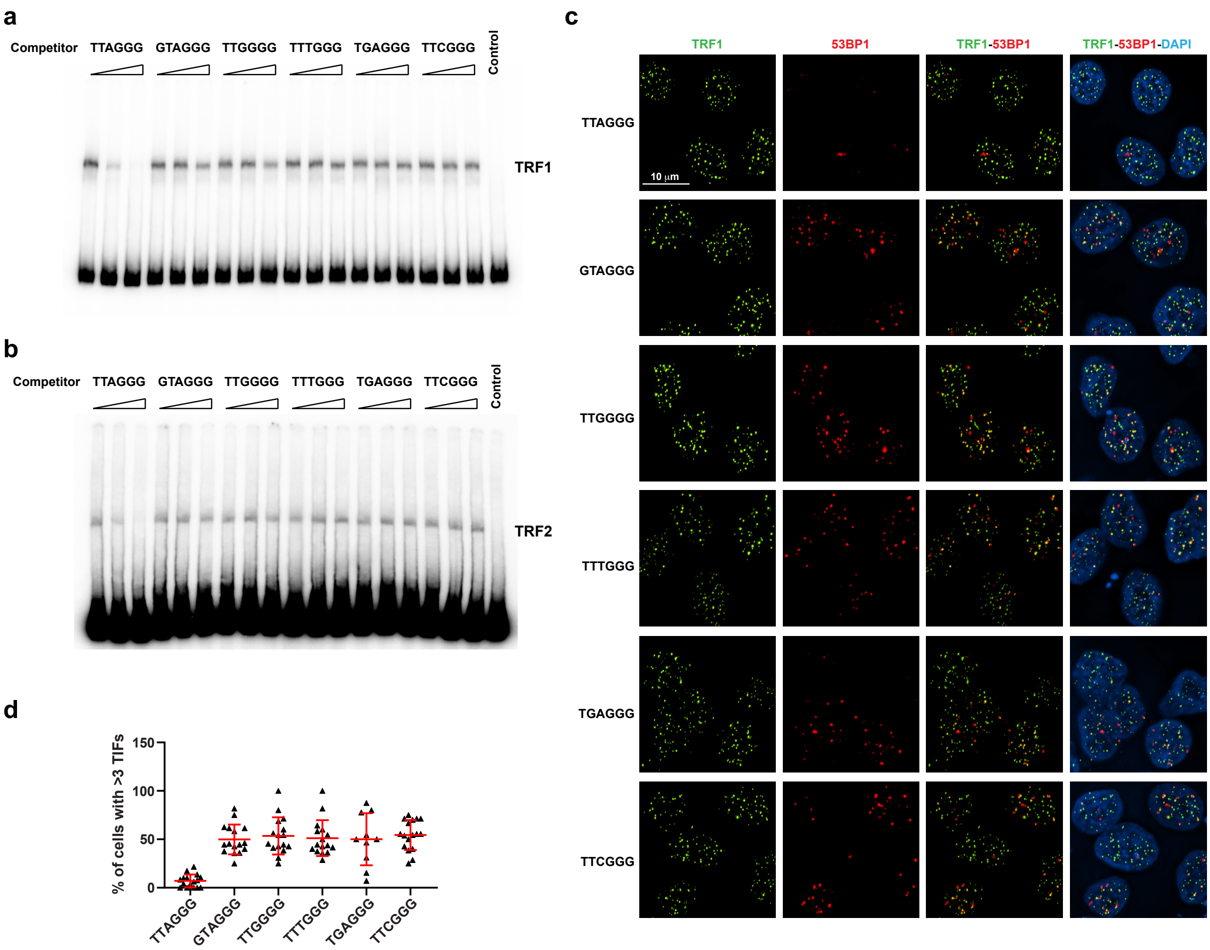

Figure S14: TVSs disrupt the binding of shelterin complex at telomere. a. Gel mobility shift assay[42] of *in vitro*-translated TRF1 and ^{32}P-labeled DNA fragment containing canonical telomere repeats sequence in the presence of competitive DNA fragments with either canonical telomere repeats or TVSs. Representative results from one of the two independent experiments are shown. **b.** Gel mobility shift assay of *in vitro*-translated TRF2 and ^{32}P-labeled DNA fragment containing canonical telomere repeats sequence in the presence of competitive DNA fragments with either canonical telomere repeats or TVSs. Representative results from one of the two independent experiments are shown. **c.** Telomere dysfunction induced foci (TIFs) in the WA18 (hTR$^{-/-}$) embryonic stem cells transduced with lentivirus co-expressing a puromycin resistance cassette and hTRs encoding either canonical telomere repeat or TVSs as indicated. Lentivirus transduced cells were subjected to puromycin selection for 48 hours (except for the "TGAGGG" variant), and then immunostaining using anti-TRF1 and anti-53BP1 antibodies to identify TIFs. Due to the toxicity of "TGAGGG", the immunostaining was done 3 days after lentivirus transduction without puromycin selection. **d.** Quantification of TIFs in WA18 hTR-/- cells expressing different hTRs. Each dot on the plot represents value obtained from one image, and bars represent mean values ± SD. About 16 images (8-15 cells per image) from two independent experiments were taken randomly and examined. In sum, >120 cells per line were analyzed for TIFs. All image files were randomly assigned coded names to allow blinded scoring for spots co-localization. The lower number of TIF in cells expressing "TGAGGG" is likely due to no enrichment of cells using puromycin selection. Therefore, a large proportion of cells likely did not uptake the lentivirus expressing the mutant hTR containing TGAGGG" variant sequence.

P14

ACGACCCCCAACCCCTGCCCAGCGACCCTCTGTGCGCCTGCGCGGGCGCGGTGCGCCTGTGTGCGCCTGCGCGGGCGCGGTGCGCCTGTGTGCGCCTGCGCGGGCGCGGTGCGCCTGTGTGCGCCTGCGCGGGCGCGGTGCGCCTGTGTG
CGCCTGCGCGGGCGCGGTGCGCCTCTCTGCGACTGCGCGGGCGCGGTGCGCCTCTCTGCGCCTGCGCGGGCGCGGTGCGCCTCTCTGCGCCTGCGCGGGCGCGGTGCGCCTCTCTGCGCCTGCGCGGGCGCGGTGCGCCTCTCTGCGCCT
GCGCGGGCGCGGTGCGCCTCTCTGCGCCTGCGCGGGCGCGGTGCGCCTCTCTGCGCCTGCGCGGGCGCGGTGCGCCTCTCTGCGCCTGCGCGGGCGCGGTGCGCCTCTCTGCGCCTGCGCGGGCGCGGTGCGCCTCTCTGCGCCTGCGCG
GGCGCGGTGCGCCTCTCTGCGCCTGCGCGGGCGCGGTGCGCCTCTCTGCGCCTGCGCGGGCGCGGTGCGCCTCTCTGCGCCTGCGCGGGGCGCGGTGCGCCTCTCTGCGCCTGCGCGGGCGCGGTGCGCCTCTCTGCGCCTGCGCGGGCG
CGGTGCGCCTCTCTGCGCCTGCGCGGGCGCGGTGCGCCTCTCTGCGCCTGCGCGGGCGCGGTGCGCCTCTCTGCGCCTGCGCGGGGCGCGGTGCGCCTCTCTGCGCCTGCGCGGGCGCGGTGCGCCTCTCTGCGCCTGCGCGGGGCGCGG
TGCGCCTCTCTGCGCCTGCGCGGGCGCGGTGCGCCTCTCTGCGCCTGCGCGGGCGCGGTGCGCCTCTCTGCGCCTGCGCGGGCGCGGTGCGCCTCTCTGCGCCTGCGCGGGCGCGGTGCGCCTCTCTGCGCCTGCGCGGGCGCGGTGCGC
CTCTCTGCGCCTGCGCGGGCGCGGTGCGCCTCTCTGCGCCTGCGCGGGCGCGGTGCGCCTCTCTGCGCCTGCGCGGGCGCGGTGCGCCTCTCTGCGCCTGCGCGGGCGCGGTGCGCCTCTCTGCGCCTGCGCGGGCGCGGTGCCCTTTGC
GACTAGGAGCTGCGTTCTCTTCACCACAGACCCGGGCGGGTGGGCCACGGGCACCGCGAAGGCGGAGCTGCGTTTTGCTGAGCACACACCCGGTGGACACCGTAAAGGCGAAGCAGCATTCTCCTCAGGACAGAGTTTGGGGGCACTGCC
TCGCTTTGGGACCCCTCGGGGCCGCATCGACGGTGAATAAAATACTTCCTGTTTGCAGCACTGAATACTCAAAGTCAGACACGAGTTAGGCTTCGGCTTAGGGTTAGGGGTTAGGGGTTAGGGCTAGGGCTAGGGCTAGGGCTAGGGTTA
GGGTTTTAGGGTTAGGGTTTGGGTCGGGGTCGGGGTCGGGTTCGGGGTGGGGGTCGGGGGTCGGGGGTCGGGGGTCGAGGGTGAGGGTGAGGGGTTAGGGTTAGGGTTGGGGTTGGGGTTGGGGTTCGGGGTTCGGGGTTCGGGGTTCGG
GTTCGGGTTCGGGTTAGGGTCAGGGTCACGGTCAGGGTCAGGGTCAGGGTTAAGGGTTAGGGTTAAGGGTTAGGGGTTAGGGGTTAGGGGTTCGGGTTAGGGTTAGGGTAAAGGGTTAGGGTTACGGTTAGGGTTAGGGTTAGGGTTAGG
GGTTAGGGTTAGGGGTTAGGGTTAGGGTTAGGGTTAGGGTTCGGGTAGGGTTAGGGTTAGGGGTTAGGGTTAGGGTAGGGTTAGGGTTAGGGTTAGGGTTAGGGTTAGGGTTAGGGTAGGGTTACGGTTAGGGTTAGGGTTAGGGTTAGG
GTTAGGGTAGGGTTAGGG
TTAGGGTTAGGGTTAGGGTTAGGGTTAGGGTTAGGGTTAGGGTTAGGGTTAGGGTTAGGGTTAGGGTTAGGGTTAGGGTTAGGGTTAGGTTAGGGTTAGGGTTAGGGTTAGGGTTAGGGTTAGGGTTAGGGTTAGGTTAGGGTTAGGGTT
AGGGTTAGGGTTAGGGTTAGGGTTAGGGTTAGGTTAGGGTTAGGGTTAGGGTTAGGGTTAGGGTTAGGGTTAGGGTTAGGGTTAGAGGGTTAGAGGGTTAGAGGGTTAGAGGGTTAGAGTTAGGGTTAGGGTTAGGGTTAGGGTTAGGGT
TAGGGTTAGGGTTAGGGTTAGGGTTAGGGTTAGGGTTAGGGTTAGGGTTAGGGTTAGGGTTAGGGTTAGGGTTAGGGTTAGGGTTAGGGTTAGGGTTAGGGTTTAGGGTTTGGTTAGGGTTAGGGTTAGGGTTAGGGTTAGGGTTAGGGT
TAGGGT
TAGGGTAGGGGTTAGGGTTAGGGTTAGGGTTAGGGTTAGGGTTAGGGTAGGGTTAGGGTTAGGGTTAGGGTTAGGGTTAGGGGTAGGGTTAGGGTTAGGGTTAGGGTTAGGGTTAGGGTTAGGGTTAGGGTTGGGTTA
GGTTAGGGGTTGGGGTTAGGGTTAGGTTTGGGTTAGGGTTAGGGTTAGGGTTAGGGTTAGGGTTAGGGTTAGGGTTAGGGTTAGGGTTAGGGTTAGGGTTAGGGGTTAGGGTTAGGGTTAGGGTTAGGGTTAGGGTTAGGGTTAGGGTT
AGGGTTGGGTTAGGGTTAGGGTTAGGGTTGGGTTAGGGTTAGGGTTAGGGTTAGGGTTAGGGTTAGGGTTAGGGTTAGGGTTAGGGTTAGGGTTAGGGGTTAGGGTTAGGGTTAGGGTTAGGGTTAGGGTTGGGTTAGGGGTTG
GGTTAGGGTTAGGGTTAGGGTTAGGGTTAGGGTTAGGGTTTGGGTTAGGGTTAGGGTTGGGTTAGGGTTAGGGTTAGGGTTAGGGTTAGGGTTAGGGTTAGGGTTGGGTTAGGGTTTGGGGTTAGGGTTAGGGTTTGG
GTTAGGGTTAGGGTTAGGGGGTAGGGTTAGGGTTGGGGTTGGGGTTGGGTTAGGGTTGGGGTGGGGGTTAGGGTGGGGTTTGGGTTAGGGTTTGGGGTTAGGGTTGGGGGTTGGGTTGGGGTTAGGGTTAGGGTTGGGGTGGGGGTTGGG
TTAGGGGTTGGGGTGTGGGTTAGGGGTTAGGGTTAGGGTTTGGGTTTGGGGTTAGGGTTGGGTTAGGGTTAGGGTTAGGGTTGGGTTTGGGTTAGGGTTGGGTTAGGGTTGGGTTAGGGTTAGGGTTAGGGTTTGGGTTAGGGTTAGGGT
TTGGGTTAGGGTTGGGTTAGGGTTAGGGTTAGGGTTAGGGTTAGGGTTAGGGGTTAGGGTTAGGGTTAGGGTTAGGGTTAGGGTTAGGGTTAGGGTTAGGGTTAGGGTTAGGGTTAGGGTTAGGGTTAGGGTTAGGGTTAGGGTTAGGGG
TTAGGG
TTAGGG
TTAGGG
GTTAGGGTTAGGGGTTAGGGTTAGGGTTAGGGTTAGGGTTAGGGTTAGGGTTAGGGTTAGGGTTAGGGTTAGGGTTAGGGTTAGGGTTAGGGTTAGGTGGGTTAGGGTTAGGGTTAGGGGGGTTAGGGTTAGGGTTAGGGTTAGGGTTAG
GGTTAGGGTTAGGGTGGGTTAGGGTTAGGGTTAGGGTTGGTAGGGTTAGGGTTGGGTTAGGGTTAGGGTTAGGGTTAGGGTTAGGGGTTAGGGTTAGGGTTAGGGTTAGGGTTAGGGTTAGGGTTAGGGTTAGGGTTAGGGTTA
GGGTTAGGGTTAGGGTTAGGGGTTAGGGTTAGGGTTAGGGTTAGGGTTAGGGTTTGGGTTAGGGTTAGGGTTAGGGTTAGGGTTAGGGTTAGGGTTAGGGTTAGGGTTAGGGTTAGGGTTAGGGTTAGGGTTAGGGTTAGGGTTAGGGTT
AGGGTTAGGGTTAGGGTTAGGGTTAGGGTTTGGGTTAGGTTAGGGTTAGGGTTAGGGGTTAGGGTTAGGGTTAGGGTTAGGGTTAGGTTAGGGTTAGGGTTAGGGTTAGGGTTAGGGTTAGGGTTAGGGTTAGGGTTAGGGTTAGGGGTT
GGGTTAGGGTTAGGGTTAGGGTTTGGGTTAGGGTTAGGGTTAGGGTTAGGGTTAGGGGTTAGGGTTAGGGTTAGGGTTAGGGTTAGGGTTAGGGTTAGGGTTAGGGTTAGGGTTAGGGTTAGGGTTAGGGTTAGGGTTAGGGTTAGGGTT
AGGGTTAGGGTTAGGGTTAGGGTTAGGGTTAGGGTTAGGGTTAGGGTTAGGGTTAGGGTTAGGGTTAGGGTTAGGGTTAGGGTTAGGGTTAGGGTTAGGGTTGGGTTAGGGTTAGGGTTAGGGTTAGGGTTAGGGTTAGGTTAGGGTTAG
GGTTAGGGTTGGGTTAGG
GTTAGG
GTTAGG
GTTAGG
GTTAGGGGTTAGGGTTAGGGTTAGGGTTAGGGTTAGGGTTAGGGTTAGGGTTAGGTTGGGTTAGGGTTAGGGTTAGGGTTAGGGTTAGGGTTAGGGTTAGGGTTAGGGTTAGGGTTAGGGTTAGGGTTAGGGTTAGGGTTAGGGTTAGGG
TTAGGGTTAGGGTTAGGGTTAGGGTTAGGGTTAGGGTTAGGGTTAGGGTTAGGGTTAGGGTTAGGGTTAGGGTTAGGGTTAGGGTTAGGGTTAGGGTTAGGGTTAGGGTAGGGTTAGGGTTAGGGTTAGGGTTAGGGTTAGGGT
TAGGGT
TAGGGTTAGGGTTAGGGTTAGGGTTAGGGTTAGGGTTAGGGTTAGGGTTAGGGTTAGGGTTAGGGTTAGGGTTAGGGTTAGGGTTAGGGTTAGGGTTAGGGTTAGGTTAGGGTTAGGGTTAGGGTTAGGGTTAGGGTTAGGGTT
AGGGTT
AGGGTTAGGGTTAGGGTTAGGGTTAGGGTAGGTTAGGGTTAGGGTTAGGGTTAGGGTTAGGGTTAGGGTTAGGGTTAGGGTTAGGGTTAGGTTAGGGTTAGGGTTAGGGTTAGGGTTAGGGTTAGGGTTAGGGTTAGGGTTAGGGTTAGG
GTTAGGGTTAGGGTTAGGGTTAGGGTTTAGGGTTAGGGTTAGGGTTAGGGTTAGGGTTAGGGTTAGGGTTAGGGTTAGGGTTAGGGTTAGGGTTAGGGTTAGGGTTAGGGTTAGGGTTAGGGTTAGGGTTAGGGTTAGGGTTAG
GGTTAGGGTTAGGGTTAGGGTTAGGGTTAGGGTTAGGGTTAGGGTTAGGGTTAGGGTTAGGGTTAGGGTTAGGGTTAGGGTTAGGTTAGGGTTAGGGTTAGGGTTAGGGTTAGGGTTAGGGTTAGGGTTAGGGTTAGGGTTAGG
GTTAGGGTTAGGGTTAGGGTTAGGGTTAGGGTTAGGGTTAGGGTTAGGGTTAGGGTTAGGGTTAGGGTTAGGGTTAGGGTTAGGGTTAGGGTTAGGGTTAGGTTAGGGTTAGGGTTAGGGTTAGGGTTAGGTTAGGGT
TAGGGTTAGGGTTAGGGTTAGGGTTAGGGTTAGGGTTAGGTTAGGGTTAGGGTTAGGGTTAGGGTTAGGGTTAGGGTTAGGGTTAGGGTTAGGGTTAGGGTTAGGGTTAGGGTTAGGGTATAGGGTTAGGGTTAGGGT
TAGGGTTAGGGTTAGGGTTAGGGTTAGGGTTAGGGTTAGGGTTAGGGTTAGGGTTAGGGTTAGGGTTAGGGTTAGGGTTAGGGTTAGGGTTAGGGGTTAGGGTTAGGGTTAGGGTTAGGGTTAGATCGAATACTCGGGATGCCA
GATGCACGGAGCAG

Figure S15: The sequence of a typical telomere-containing PacBio HiFi sequencing read of Chr. 7q obtained from P14. The first 1kb of canonical telomere repeats next to the sub-telomeric region is highlighted in red. The telobait sequence and the barcode within are highlighted in green and blue, respectively.

P13

ACGACCCCCAACCCCTGCCCAGCGACCCTCTGTGCGCCTGCGCGGGCGCGGTGCGCCTGTGTGCGCCTGCGCGGGCGCGGTGCGCCTGTGTGCGCCTGCGCGGGCGCGGTGCGCCTGTGTGCGCCTGCGCGGGCGCGGTGCGCCTCTCTG
CGCCTGCGCGGGCGCGGTGCGCCTCTCTGCGCCTGCGCGGGCGCGGTGCGCCTCTCTGCGCCTGCGCGGGCGCGGTGCGCCTCTCTGCGCCTGCGCGGGCGCGGTGCGCCTCTCTGCGCCTGCGCGGGCGCGGTGCGCCTCTCTGCGCCT
GCGCGGGCGCGGTGCGCCTCTCTGCGCCTGCGCGGGCGCGGTGCGCCTCTCTGCGCCTGCGCGGGCGCGGTGCGCCTCTCTGCGCCTGCGCGGGCGCGGTGCGCCTCTCTGCGCCTGCGCGGGCGCGGTGCGCCTCTCTGCGCCTGCGCG
GGCGCGGTGCGCCTCTCTGCGCCTGCGCGGGCGCGGTGCGCCTCTCTGCGCCTGCGCGGGCGCGGTGCGCCTCTCTGCGCCTGCGCGGGCGCGGTGCGCCTCTCTGCGCCTGCGCGGGCGCGGTGCGCCTCTCTGCGCCTGCGCGGGCGC
GGTGCGCCTCTCTGCGCCTGCGCGGGCGCGGTGCGCCTCTCTGCGCCTGCGCGGGCGCGGTGCGCCTCTCTGCGCCTGCGCGGGCGCGGTGCGCCTCTCTGCGCCTGCGCGGGCGCGGTGCGCCTCTCTGCGCCTGCGCGGGCGCGGTGC
GCCTCTCTGCGCCTGCGCGGGCGCGGTGCGCCTCTCTGCGCCTGCGCGGGCGCGGTGCGCCTCTCTGCGCCTGCGCGGGCGCGGTGCCTTTGCGACTAGGAGCTGCGTTCTCTTCACCACAGACCCGGGCGGGTGGGCCACGGGCACCGC
GAAGGCGGAGCTGCGTTTTGCTGAGCACACACCCGGTGGACACCGTAAAGGCGAAGCAGCATTCTCCTCAGGACAGAGTTTGGGGGCACTGCCTCGCTTTGGGACCCCTCGGGGCCGCATCGACGGTGAATAAAATACTTCCTGTTTGCA
GCACTGAATACTCAAAGTCAGACACGAGTTAGGCTTCGGCTTAGGGTTAGGGGTTAGGGGTTAGGGCTAGGGCTAGGGCTAGGGCTAGGGTTAGGGTTTTAGGGTTAGGGTTTGGGTCGGGGTCGGGGTCGGGTTCGGGGTGGGGGTCGG
GGGTCGGGGGTCGGGGGTCGAGGGTGAGGGTGAGGGGTTAGGGTTAGGGTTGGGGTTGGGGTTGGGGTTCGGGGTTCGGGGTTCGGGGTTCGGGTTCGGGTTCGGGTTAGGGTCAGGGTCACGGTCAGGGTCAGGGTCAGGGTTAAGGGT
TAGGGTTAAGGGTTAGGGGTTAGGGGTTAGGGGTTCGGGTTAGGGTTAGGGTAAAGGGTTAGGGTTACGGTTAGGGTTAGGGTTAGGGTTAGGGGTTAGGGTTAGGGGTTAGGGTTAGGGTTCGGGTAGGGTTCGGGTTAGGGGTTCGGG
TTAGGGGTTAGGGTTAGGGTTAGGGTTAGGGTTAGGGTTAGGGTTAGGGTTTAGGGGTTAGGGGTTAGGGTTAGGGTTAGGGCTAGGGCTAGGGCTAGGGCTAGGGCTAGGGCTAGGGCTAGGGTTTAGGGTTTAGGGTTAGGGTTAGGG
TTAGGGTTAGGGTTAGGGTAGGGTAGGGTAGGGTAGGGTAAGGGTAGGGTTAGGGTTAGGGTTAGGGTTAGGGTTAGGGTTAAGGGTTAAGGGTTAGGGTTAGGGTTAGGGTTAGGGTTAGGGTTAGGGTTGGGTTAGGGTTAGGGTTAG
GGTTAGGGTTAGGGTTAGGGTTGGGTTAGGGTTGGGTTAGGGTTAGGGTTAGGGTTAGGGTTAGGGTTAGGGTTAGGGTTAAGGGTTAGGGTTAGGGTTAGGGTTAGGGTTAAGGGTTAGGGTTAGGGTTGGGTTAGGGTTAGGGTTAGG
GTTAGGGTTAAGGGTTAAGGGTTGGGGTGGGGGTGGGGGTGGGGGTGGGGGTGGGGGTGGGGTTGGGGTTGGGGTTGGGGTTGGGGTTGGGGGTTGGGGTTGGGGTTGGGGTTGGGGTTGGGGTTAAGGGTTAGGGTTAGGGTTAGGGTT
AGGGTTAGGGTTAGGGTTAGGGTAGGGTTAGGGTTAGGGTTAGGGTTAGGGTAGGGTTGGGTTAGGGTTAGGGTTAGGGTTAGGGTTAGGGGTTAGGGGTTAGGGTTAGGGTTAGGGTTAGGGTTAGGGTTAGGGTTAGGGGTTAGGGGT
TAGGGGTTAGGGTTAGGGTTAGGGTTTGGGGTTAGGGTTAGGGTTAGGGTTAGGGTTTAGGGTTTAGGGTTAGGGTTAGGGTTAGGGTTAGGGGTTGGGGTTGGGGTTGGGGTTAGGGTTAGGGTTAGGGTTAGGGTTAGGGTAGGGTAG
GGTTAGGGTAGGGTTAGGGTTAGGGTTAGGGTTAGGGTTAGGGTTAGGGTTAAGGGTTAAGGGTTAGGGTTAGGGTTAGGGGTTAGGGGTTAGGGGTTAGGGTTAGGGTTAGGGTTAGGGTTAGGGTTAGGGTTAGGGGTTAGGGGTTAG
GGTTGGGGTTGGGGTTGGGGTTGGGGTTGGGGTTGGGGTTGGGGTTGGGGTTAGGGGTTAGGGGTTAGGGTTGGGGTTGGGGTTGGGGTTGGGGTTGGGGTTGGGGTTGGGGTGAGGGTGAGGGTGAGGGTGAGGGTGAGGGTGAGGGTG
AGGGTTTTAGGGTTTTAGGGTTTTAGGGTTTTAGGGTTTTAGGGTTTTAGGGTTTTAGGGTTAGGGTTAGGGTTAGGGTTAGGGTTAGGGTGTTAGGGTTAGGGGTTAGGGTTAGGGGGTTAGGGTTAGGGTTAGGGTTAGGGTTAGG
GTTAGGGTTAGGGTTAGGGGTTAGGGGTTAGGGGTTAGGGTTAGGGTTAGGGTTAGGGTTAGGGTTAGGGTTAGGGTTAGGGTTAGGGTTAGGGTTAGGGTTAGGGTTAGGGTTAGGGTTAGGGTTAGGGTTAGGGTTAGGGTTAGGGTT
AGGGTTAGGGTTAGGGTTAGGGTTAGGGTTAGGGTTAGGGTTAGGGTTAGGGTTAGGGTTAGGGTTAGGGTTAGGGTTAGGGTTAGGGTTAGGGGGGTTAGGGGTTAGGGTTAGGGTTAGGGTTAGGGTTAGGGTTAG
GGTTAG
GGTTAGGGTTGGGTTAGG
GTTAGG
GTTAGG
GTTAGGGTTAGGGTTAGGGTTAGGGTTAGGGTTAGGGTTAGGGTAGGGTTAGGGTTAGGGTTAGGGTTAGGGTTAGGGTTAGGGTTAGGGTTAGGGTTAGGGTTAGGGTTAGGGTTAGGGTTAGGGTTAGGGTGGGTTAGGGTTAGGGTT
AGGGTT
AGGGTTAGGGTTAGGGTTAGGGTTGGGTTA
GGGTTAGGGTTTGGGTTA
GGGTTA
GGGTTA
GGGTTAGGGTTAGGGTTTGGGTTAGGGTTAGGGTTAGGGTTAGGGTTAGGGTTAGGGTTAGGGTTAGGGTTAGGGTTAGGGTTAGGGTTAGGGTTGGGTTAGGGTTAGGGTTAGGGTTAGGGTTAGGGTTAGGGTTAGGGTTAGGGTTAG
GGTTAGGGTTAGGGTTAGGGTTAGGGTTAGGGTTAGGGTTAGGGTTAGGGTTAGGGTTAGGGTTAGGGTTAGGGTTAGGGTTAGGTTAGGGTTAGGGTTAGGGTTAGGGTTAGGGTTAGGGTTAGGGTTAGGGTTAGGGTTAGG
GTTAGGGTTAGGGTTAGGGTTAGGGTTAGGGTTAGGGTTAGGGTTAGGGTTAGGGTTAGGGTTAGGGTTAGGGTTAGGGTTAGGGTTAGGGTTAGGGTTTAGGGTTAGGGTTAGGGTTAGGGTTAGGGTAGGGTTAGG
GTTAGG
GTTAGG
GTTAGG
GTTAGG
GTTAGGGGTTAGGGTTAGGGTTAGGGTTAG
GGTTAG
GGTTAG
GGTTAG
GGTTAG
GGTTAGGGTTAGGGTTAGGGTTAGGGTTAGGGTTAGGGTTAGGGTTAGGGTTAGGGTTAGGGTTAGGGTTGGGTTAGGGTTAGGGTTAGGGTTAGGGTTAGGGTTAGGGTTAGGGTTAGGGTTAGGGTTAGGGTTAGGGTTAGG
GTTAGGGTTAGATCGAATTGGATT
GATGCCAGATGCACGGAGCAGAATT

Figure S16: The sequence of a typical telomere-containing PacBio HiFi sequencing read of Chr. 7q obtained from P15. The first 1kb of canonical telomere repeats next to the sub-telomeric region is highlighted in red. The telobait sequence and the barcode within are highlighted in green and blue, respectively.

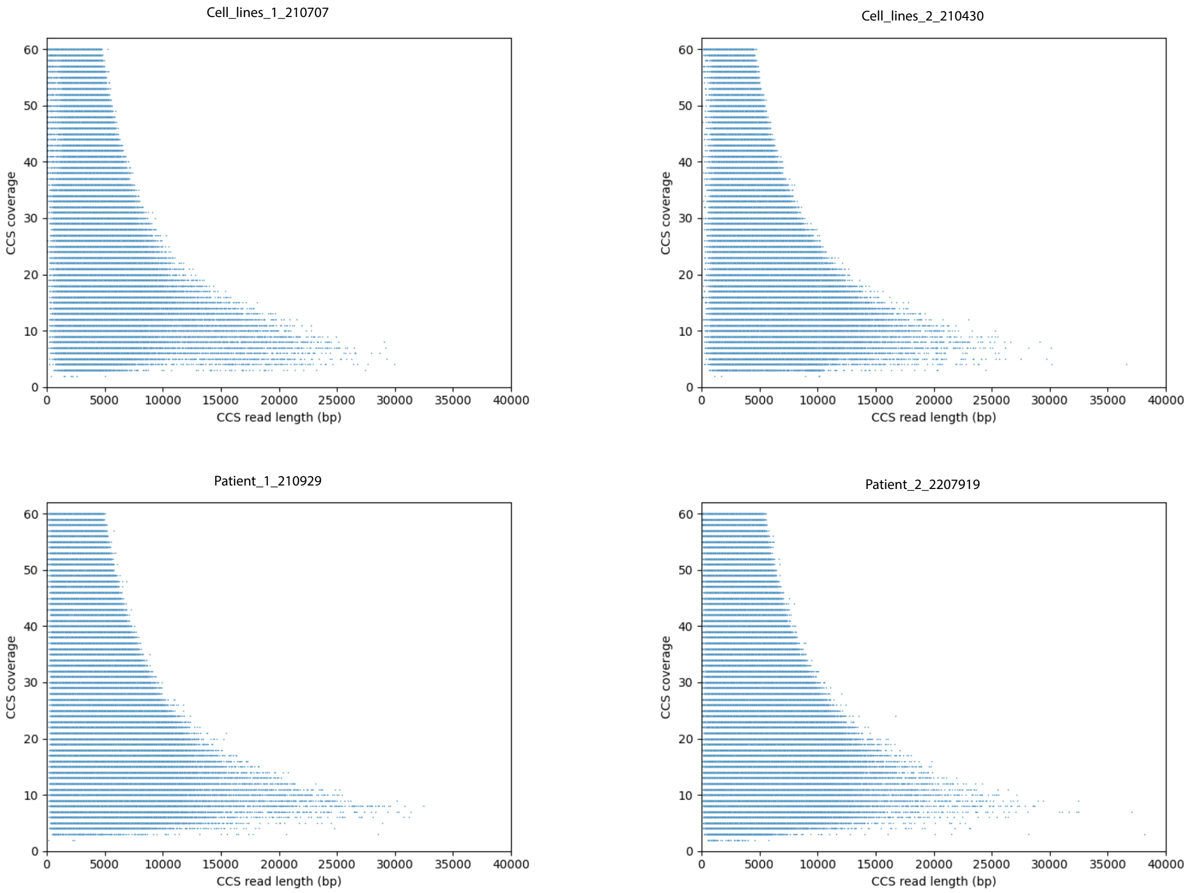

Figure S17: CCS read coverage and length for all four PacBIo Hi-Fi sequencing runs presented in this paper.

Un-cropped immunostaining raw data for Figure S14c

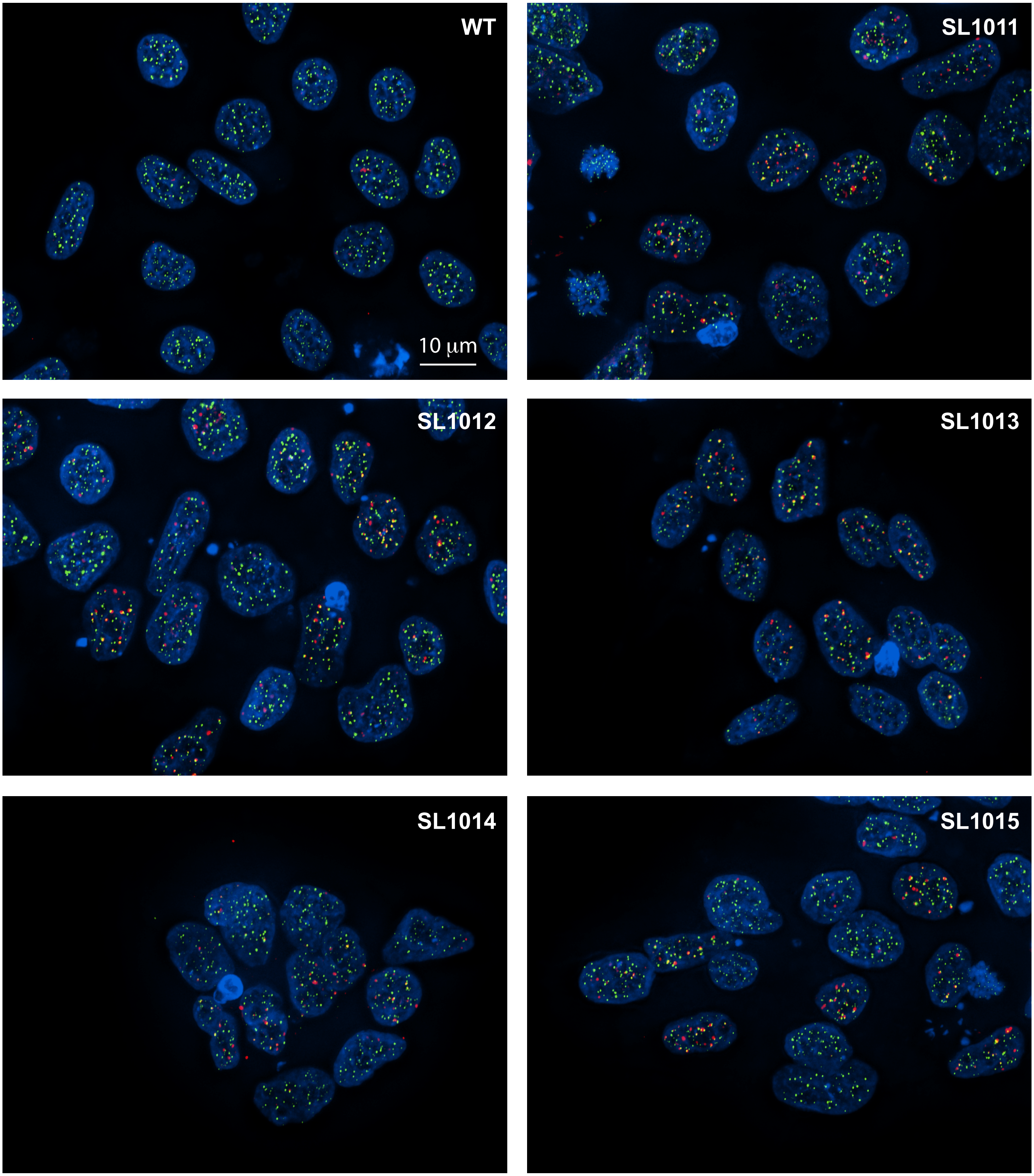

Supplement: Supplementary file 3 — Supplementary information [file 41467_2023_35823_MOESM3_ESM.pdf]
