# Supplementary material for: High-throughput telomere length measurement at nucleotide resolution using the PacBio high fidelity sequencing platform
Source: Nat Commun. 2023 Jan 17;14:281. doi: 10.1038/s41467-023-35823-7 (PMC9845338; doi:10.1038/s41467-023-35823-7)
Supplement: Supplementary file 9 — Dataset 6 [file 41467_2023_35823_MOESM9_ESM.pdf]

Axil Scientific Pte Ltd
2 Tukang Innovation Grove, #06-01, JTC MedTech Hub,
Singapore 618305
T: +65 6775 7318
F: +65 6775 7211
E: sequencing@axilscientific.com

Apical Scientific Sdn Bhd
Lot 7-1 to 7-4, Jalan SP 2/7, Taman Serdang Perdana,
Seksyen 2, 43300 Seri Kembangan, Selangor, Malaysia
T: +603 8943 3252
F: +603 8943 3243
E: sequencing@apicalscientific.com

1st BASE
YOUR RESEARCH, OUR BUSINESS
www.base-asia.com

CUSTOMER INFORMATION

Name	Poon Lai Fong	Order ID	892
Address		Date Sample Received	11-Nov-2022
Email	gmsplf@nus.edu.sg; laifong.poon@duke-nus.edu.sg	Report Date	22-Nov-2022

METHODOLOGY

Twenty-four short tandem repeat (STR) loci plus the gender determining locus, Amelogenin, were amplified using the commercially available GenePrint® 24 System from Promega. The sample was processed using the Applied Biosystems™ DNA Analyzer. Data were analyzed using GeneMapper® v4.0 software (Applied Biosystems™). Appropriate positive and negative controls were run and confirmed for each sample submitted.

SAMPLE INFORMATION

**Sample Name**	**Cell Line Designation**
293T	293T ATCC #CRL-3216

STR PROFILING RESULTS

	**Test Result for Sample**				**ATCC Reference Database Profile**			
**LOCI**	**293T**				**293TEmbryonic Kidney CellsHuman**			
Amelogenin	X				X			
D3S1358	15	16	17					
D1S1656	15	17.3		1				
D2S441	11	15						
D10S1248	13	14						
D13S317	12	14			12	14		
Penta E	7	15						
D16S539	9	13			9	13		
D18S51	17	18						
D2S1338	19							
CSF1PO	11	12			11	12		
Penta D	9	10						
TH01	7	9.3			7	9.3		
vWA	16	19			16	19		
D21S11	28	30.2	37.2					
D7S820	11				11			
D5S818	8	9			8	9		
DYS391								
TPOX	11				11			
D8S1179	11	12						
D12S391	19	20	21					
D19S433	17	18						
FGA	22	23						
D22S1045	16	17						

Number of shared alleles between sample and database profile:	15
Total number of alleles in the database profile:	15
Percent match between the submitted sample and the database profile:	100%

The allele match algorithm compares the loci highlighted in grey only (8 core loci plus amelogenin).

EXPLANATION OF TEST RESULTS

☐ The submitted sample profile is human, but not a match for any profile in the STR database.

☑ The submitted sample profile showed 80% to 100% match for the following ATCC human cell line(s) in the STR database (8 core loci plus Amelogenin): **293TEmbryonic Kidney CellsHuman**

☐ The submitted profile is similar to the following ATCC human cell line(s):

☐ The submitted sample is a mixture. Multiple peaks are observed in the STR profiling results.

ADDITIONAL INFORMATION: Comparative Data Output from ATCC STR Profile Database

% Match	ATCC Number	Designation	D5S818	D13S317	D7S820	D16S539	vWA	TH01	AMEL	TPOX	CSF1PO
100	CRL-3216	293TEmbryonic Kidney CellsHuman	8,9	12,14	11	9,13	16,19	7,9.3	X	11	11,12

For alternate database, you may visit https://www.dsmz.de/services/services-human-and-animal-cell-lines/online-str-analysis.html

End of report

Fragment Analysis
- STR Profiling
Cell Line Authentication

Axil Scientific Pte Ltd
2 Tukang Innovation Grove, #06-01, JTC MedTech Hub,
Singapore 618305
T: +65 6775 7318
F: +65 6775 7211
E: sequencing@axilscientific.com

Apical Scientific Sdn Bhd
Lot 7-1 to 7-4, Jalan SP 2/7, Taman Serdang Perdana,
Seksyen 2, 43300 Seri Kembangan, Selangor, Malaysia
T: +603 8943 3252
F: +603 8943 3243
E: sequencing@apicalscientific.com

1st BASE
YOUR RESEARCH, OUR BUSINESS
www.base-asia.com

CUSTOMER INFORMATION

Name	Poon Lai Fong	Order ID	892
Address		Date Sample Received	11-Nov-2022
Email	gmsplf@nus.edu.sg; laifong.poon@duke-nus.edu.sg	Report Date	22-Nov-2022

METHODOLOGY

Twenty-four short tandem repeat (STR) loci plus the gender determining locus, Amelogenin, were amplified using the commercially available GenePrint® 24 System from Promega. The sample was processed using the Applied Biosystems™ DNA Analyzer. Data were analyzed using GeneMapper® v4.0 software (Applied Biosystems™). Appropriate positive and negative controls were run and confirmed for each sample submitted.

SAMPLE INFORMATION

**Sample Name**	**Cell Line Designation**
HeLa	HeLa ATCC#CCL-2

STR PROFILING RESULTS

	**Test Result for Sample**				**ATCC Reference Database Profile**			
**LOCI**	**HeLa**				**HeLaCervical AdenocarcinomaHuman**			
Amelogenin	X				X			
D3S1358	15	18						
D1S1656	12	15						
D2S441	10	11						
D10S1248	13	15						
D13S317	12	13.3			12	13.3		
Penta E	7	17						
D16S539	9	10			9	10		
D18S51	15.3	16						
D2S1338	17							
CSF1PO	9	10			9	10		
Penta D	8	15						
TH01	7				7			
vWA	16	18			16	18		
D21S11	27	28						
D7S820	7.3	8	12		8	12		
D5S818	11	12			11	12		
DYS391								
TPOX	8	12			8	12		
D8S1179	12	13						
D12S391	20	25						
D19S433	13	14						
FGA	18	21						
D22S1045	16	17						

Number of shared alleles between sample and database profile:	16
Total number of alleles in the database profile:	16
Percent match between the submitted sample and the database profile:	100%

The allele match algorithm compares the loci highlighted in grey only (8 core loci plus amelogenin).

EXPLANATION OF TEST RESULTS

☐ The submitted sample profile is human, but not a match for any profile in the STR database.

☑ The submitted sample profile showed 80% to 100% match for the following ATCC human cell line(s) in the STR database (8 core loci plus Amelogenin): **HeLaCervical AdenocarcinomaHuman**

☐ The submitted profile is similar to the following ATCC human cell line(s):

☐ The submitted sample is a mixture. Multiple peaks are observed in the STR profiling results.

ADDITIONAL INFORMATION: Comparative Data Output from ATCC STR Profile Database

% Match	ATCC Number	Designation	D5S818	D13S317	D7S820	D16S539	vWA	TH01	AMEL	TPOX	CSF1PO
100	CCL-2	HeLaCervical AdenocarcinomaHuman	11,12	12,13.3	8,12	9,10	16,18	7	X	8,12	9,10

For alternate database, you may visit https://www.dsmz.de/services/services-human-and-animal-cell-lines/online-str-analysis.html

End of report

Fragment Analysis
- STR Profiling
Cell Line Authentication

Axil Scientific Pte Ltd
2 Tukang Innovation Grove, #06-01, JTC MedTech Hub, Singapore 618305
T: +65 6775 7318
F: +65 6775 7211
E: sequencing@axilscientific.com

Apical Scientific Sdn Bhd
Lot 7-1 to 7-4, Jalan SP 2/7, Taman Serdang Perdana, Seksyen 2, 43300 Seri Kembangan, Selangor, Malaysia
T: +603 8943 3252
F: +603 8943 3243
E: sequencing@apicalscientific.com

1st BASE
YOUR RESEARCH, OUR BUSINESS
www.base-asia.com

CUSTOMER INFORMATION

Name	Poon Lai Fong	Order ID	892
Address		Date Sample Received	11-Nov-2022
Email	gmsplf@nus.edu.sg; laifong.poon@duke-nus.edu.sg	Report Date	22-Nov-2022

METHODOLOGY

Twenty-four short tandem repeat (STR) loci plus the gender determining locus, Amelogenin, were amplified using the commercially available GenePrint® 24 System from Promega. The sample was processed using the Applied Biosystems™ DNA Analyzer. Data were analyzed using GeneMapper® v4.0 software (Applied Biosystems™). Appropriate positive and negative controls were run and confirmed for each sample submitted.

SAMPLE INFORMATION

**Sample Name**	**Cell Line Designation**
T24	T24 ATCC #HTB-4

STR PROFILING RESULTS

	**Test Result for Sample**				**ATCC Reference Database Profile**			
**LOCI**	**T24**				**T-24Bladder CancerHuman**			
Amelogenin	X				X			
D3S1358	9	16						
D1S1656	12	15						
D2S441	11	15						
D10S1248	14							
D13S317	12				12			
Penta E	7	10						
D16S539	9				9			
D18S51	16	18						
D2S1338	20	23						
CSF1PO	10	12			10	12		
Penta D	11	15						
TH01	6				6			
vWA	17				17			
D21S11	29							
D7S820	10	11			10	11		
D5S818	10	12			10	12		
DYS391								
TPOX	8	11			8	11		
D8S1179	14							
D12S391	18							
D19S433	13	14						
FGA	17	22						
D22S1045	16							

Number of shared alleles between sample and database profile:	13
Total number of alleles in the database profile:	13
Percent match between the submitted sample and the database profile:	100%

The allele match algorithm compares the loci highlighted in grey only (8 core loci plus amelogenin).

EXPLANATION OF TEST RESULTS

☐ The submitted sample profile is human, but not a match for any profile in the STR database.

☑ The submitted sample profile showed 80% to 100% match for the following ATCC human cell line(s) in the STR database (8 core loci plus Amelogenin): **T-24Bladder CancerHuman**

☐ The submitted profile is similar to the following ATCC human cell line(s):

☐ The submitted sample is a mixture. Multiple peaks are observed in the STR profiling results.

ADDITIONAL INFORMATION: Comparative Data Output from ATCC STR Profile Database

% Match	ATCC Number	Designation	D5S818	D13S317	D7S820	D16S539	vWA	TH01	AMEL	TPOX	CSF1PO
100	HTB-4	T-24Bladder CancerHuman	10,12	12	10,11	9	17	6	X	8,11	10,12

For alternate database, you may visit https://www.dsmz.de/services/services-human-and-animal-cell-lines/online-str-analysis.html

End of report

Fragment Analysis
- STR Profiling
Cell Line Authentication

Axil Scientific Pte Ltd
2 Tukang Innovation Grove, #06-01, JTC MedTech Hub,
Singapore 618305
T: +65 6775 7318
F: +65 6775 7211
E: sequencing@axilscientific.com

Apical Scientific Sdn Bhd
Lot 7-1 to 7-4, Jalan SP 2/7, Taman Serdang Perdana,
Seksyen 2, 43300 Seri Kembangan, Selangor, Malaysia
T: +603 8943 3252
F: +603 8943 3243
E: sequencing@apicalscientific.com

CUSTOMER INFORMATION

Name	Poon Lai Fong	Order ID	892
Address		Date Sample Received	11-Nov-2022
Email	gmsplf@nus.edu.sg; laifong.poon@duke-nus.edu.sg	Report Date	22-Nov-2022

METHODOLOGY

Twenty-four short tandem repeat (STR) loci plus the gender determining locus, Amelogenin, were amplified using the commercially available GenePrint® 24 System from Promega. The sample was processed using the Applied Biosystems™ DNA Analyzer. Data were analyzed using GeneMapper® v4.0 software (Applied Biosystems™). Appropriate positive and negative controls were run and confirmed for each sample submitted.

SAMPLE INFORMATION

Sample Name	Cell Line Designation
HCT116	HCT 116 ATCC #CCL-247

STR PROFILING RESULTS

	Test Result for Sample				ATCC Reference Database Profile			
**LOCI**	**HCT116**				**HCT 116Colon CarcinomaHuman**			
Amelogenin	X				X	Y		
D3S1358	12	18	19					
D1S1656	12	13	14					
D2S441	11	12						
D10S1248	11	14	15					
D13S317	10	12			10	12		
Penta E	12	13	14					
D16S539	11	13			11	13		
D18S51	16	17						
D2S1338	16							
CSF1PO	7	10			7	10		
Penta D	9	13						
TH01	8	9			8	9		
vWA	17	22			17	22		
D21S11	29	30						
D7S820	11	12			11	12		
D5S818	10	11			10	11		
DYS391								
TPOX	8	9			8	9		
D8S1179	10	14	15					
D12S391	17	22						
D19S433	12							
FGA	18	23						
D22S1045	14	16						

Number of shared alleles between sample and database profile:	17
Total number of alleles in the database profile:	18
Percent match between the submitted sample and the database profile:	94%

The allele match algorithm compares the loci highlighted in grey only (8 core loci plus amelogenin).

EXPLANATION OF TEST RESULTS

☐ The submitted sample profile is human, but not a match for any profile in the STR database.

☑ The submitted sample profile showed 80% to 100% match for the following ATCC human cell line(s) in the STR database (8 core loci plus Amelogenin): **HCT 116Colon CarcinomaHuman**

☐ The submitted profile is similar to the following ATCC human cell line(s):

☐ The submitted sample is a mixture. Multiple peaks are observed in the STR profiling results.

ADDITIONAL INFORMATION: Comparative Data Output from ATCC STR Profile Database

% Match	ATCC Number	Designation	D5S818	D13S317	D7S820	D16S539	vWA	TH01	AMEL	TPOX	CSF1PO
94	CCL-247	HCT 116Colon CarcinomaHuman	10,11	10,12	11,12	11,13	17,22	8,9	X,Y	8,9	7,10

For alternate database, you may visit https://www.dsmz.de/services/services-human-and-animal-cell-lines/online-str-analysis.html

End of report

Fragment Analysis
- STR Profiling
Cell Line Authentication

Axil Scientific Pte Ltd
2 Tukang Innovation Grove, #06-01, JTC MedTech Hub,
Singapore 618305
T: +65 6775 7318
F: +65 6775 7211
E: sequencing@axilscientific.com

Apical Scientific Sdn Bhd
Lot 7-1 to 7-4, Jalan SP 2/7, Taman Serdang Perdana,
Seksyen 2, 43300 Seri Kembangan, Selangor, Malaysia
T: +603 8943 3252
F: +603 8943 3243
E: sequencing@apicalscientific.com

1st BASE
YOUR RESEARCH, OUR BUSINESS
www.base-asia.com

CUSTOMER INFORMATION

Name	Poon Lai Fong	Order ID	892
Address		Date Sample Received	11-Nov-2022
Email	gmsplf@nus.edu.sg; laifong.poon@duke-nus.edu.sg	Report Date	22-Nov-2022

METHODOLOGY

Twenty-four short tandem repeat (STR) loci plus the gender determining locus, Amelogenin, were amplified using the commercially available GenePrint® 24 System from Promega. The sample was processed using the Applied Biosystems™ DNA Analyzer. Data were analyzed using GeneMapper® v4.0 software (Applied Biosystems™). Appropriate positive and negative controls were run and confirmed for each sample submitted.

SAMPLE INFORMATION

Sample Name	Cell Line Designation
IMR90	IMR-90 ATCC #CCL-186

STR PROFILING RESULTS

	Test Result for Sample				ATCC Reference Database Profile			
**LOCI**	**IMR90**				**IMR-90Lung Fibroblast Human**			
Amelogenin	X				X			
D3S1358	14	15		19				
D1S1656	11	15						
D2S441	11	13						
D10S1248	13	14						
D13S317	11	13			11	13		
Penta E	5							
D16S539	10	13			10	13		
D18S51	17							
D2S1338	19	25	29					
CSF1PO	11	13			11	13		
Penta D	5	12						
TH01	8	9.3			8	9.3		
vWA	16	19			16	19		
D21S11	30.2	31						
D7S820	9	12			9	12		
D5S818	12	13			12	13		
DYS391								
TPOX	8	9			8	9		
D8S1179	13	14						
D12S391	18	21						
D19S433	10	13						
FGA	25	26						
D22S1045	12	16						

Number of shared alleles between sample and database profile:	17
Total number of alleles in the database profile:	17
Percent match between the submitted sample and the database profile:	100%

The allele match algorithm compares the loci highlighted in grey only (8 core loci plus amelogenin).

EXPLANATION OF TEST RESULTS

☐ The submitted sample profile is human, but not a match for any profile in the STR database.

☑ The submitted sample profile showed 80% to 100% match for the following ATCC human cell line(s) in the STR database (8 core loci plus Amelogenin): **IMR-90Lung Fibroblast Human**

☐ The submitted profile is similar to the following ATCC human cell line(s):

☐ The submitted sample is a mixture. Multiple peaks are observed in the STR profiling results.

ADDITIONAL INFORMATION: Comparative Data Output from ATCC STR Profile Database

% Match	ATCC Number	Designation	D5S818	D13S317	D7S820	D16S539	vWA	TH01	AMEL	TPOX	CSF1PO
100	CCL-186	IMR-90Lung Fibroblast Human	12,13	11,13	9,12	10,13	16,19	8,9.3	X	8,9	11,13

For alternate database, you may visit https://www.dsmz.de/services/services-human-and-animal-cell-lines/online-str-analysis.html

End of report

Axil Scientific Pte Ltd
2 Tukang Innovation Grove, #06-01, JTC MedTech Hub,
Singapore 618305
T: +65 6775 7318
F: +65 6775 7211
E: sequencing@axilscientific.com

Apical Scientific Sdn Bhd
Lot 7-1 to 7-4, Jalan SP 2/7, Taman Serdang Perdana,
Seksyen 2, 43300 Seri Kembangan, Selangor, Malaysia
T: +603 8943 3252
F: +603 8943 3243
E: sequencing@apicalscientific.com

Fragment Analysis
- STR Profiling
Cell Line Authentication

1st BASE
YOUR RESEARCH, OUR BUSINESS
www.base-asia.com

CUSTOMER INFORMATION

Name	Poon Lai Fong	Order ID	892
Address		Date Sample Received	11-Nov-2022
Email	gmsplf@nus.edu.sg; laifong.poon@duke-nus.edu.sg	Report Date	22-Nov-2022

METHODOLOGY

Twenty-four short tandem repeat (STR) loci plus the gender determining locus, Amelogenin, were amplified using the commercially available GenePrint® 24 System from Promega. The sample was processed using the Applied Biosystems™ DNA Analyzer. Data were analyzed using GeneMapper® v4.0 software (Applied Biosystems™). Appropriate positive and negative controls were run and confirmed for each sample submitted.

SAMPLE INFORMATION

Sample Name	Cell Line Designation
WI-38	WI-38 ATCC #CCL-75

STR PROFILING RESULTS

	Test Result for Sample				ATCC Reference Database Profile			
**LOCI**	**WI-38**				**WI-38 Lung Fibroblast Human**			
Amelogenin	X				X			
D3S1358	16	17						
D1S1656	11	18.3						
D2S441	11							
D10S1248	13							
D13S317	11				11			
Penta E	13	14						
D16S539	11	12			11	12		
D18S51	16	18						
D2S1338	19	25						
CSF1PO	10	12			10	12		
Penta D	13							
TH01	8	9.3			8	9.3		
vWA	19	20			19	20		
D21S11	30	30.2						
D7S820	9	11			9	11		
D5S818	10				10			
DYS391								
TPOX	8				8			
D8S1179	14							
D12S391	21	23						
D19S433	13	16.2						
FGA	22	24						
D22S1045	11	17						

Number of shared alleles between sample and database profile:	14
Total number of alleles in the database profile:	14
Percent match between the submitted sample and the database profile:	100%

The allele match algorithm compares the loci highlighted in grey only (8 core loci plus amelogenin).

EXPLANATION OF TEST RESULTS

☐ The submitted sample profile is human, but not a match for any profile in the STR database.

☑ The submitted sample profile showed 80% to 100% match for the following ATCC human cell line(s) in the STR database (8 core loci plus Amelogenin): **WI-38 Lung Fibroblast Human**

☐ The submitted profile is similar to the following ATCC human cell line(s):

☐ The submitted sample is a mixture. Multiple peaks are observed in the STR profiling results.

ADDITIONAL INFORMATION: Comparative Data Output from ATCC STR Profile Database

% Match	ATCC Number	Designation	D5S818	D13S317	D7S820	D16S539	vWA	TH01	AMEL	TPOX	CSF1PO
100	CCL-75	WI-38 Lung Fibroblast Human	10	11	9,11	11,12	19,20	8,9.3	X	8	10,12

For alternate database, you may visit https://www.dsmz.de/services/services-human-and-animal-cell-lines/online-str-analysis.html

End of report

Axil Scientific Pte Ltd
2 Tukang Innovation Grove, #06-01, JTC MedTech Hub,
Singapore 618305
T: +65 6775 7318
F: +65 6775 7211
E: sequencing@axilscientific.com

Apical Scientific Sdn Bhd
Lot 7-1 to 7-4, Jalan SP 2/7, Taman Serdang Perdana,
Seksyen 2, 43300 Seri Kembangan, Selangor, Malaysia
T: +603 8943 3252
F: +603 8943 3243
E: sequencing@apicalscientific.com

CUSTOMER INFORMATION

Name	Poon Lai Fong	Order ID	892
Address		Date Sample Received	11-Nov-2022
Email	gmsplf@nus.edu.sg; laifong.poon@duke-nus.edu.sg	Report Date	22-Nov-2022

METHODOLOGY

Twenty-four short tandem repeat (STR) loci plus the gender determining locus, Amelogenin, were amplified using the commercially available GenePrint® 24 System from Promega. The sample was processed using the Applied Biosystems™ DNA Analyzer. Data were analyzed using GeneMapper® v4.0 software (Applied Biosystems™). Appropriate positive and negative controls were run and confirmed for each sample submitted.

SAMPLE INFORMATION

Sample Name	Cell Line Designation
VA13	VA13 (derived from WI-38)

STR PROFILING RESULTS

	Test Result for Sample				ATCC Reference Database Profile			
**LOCI**	**VA13**				**WI-38 VA-13 Subline 2RA Lung Human**			
Amelogenin	X				X			
D3S1358	16	17						
D1S1656	11	18.3						
D2S441	11	12						
D10S1248	13	15	16					
D13S317	11				11			
Penta E	13	14						
D16S539	11	12			11	12		
D18S51	16	18						
D2S1338	19	25						
CSF1PO	10	12			10	12		
Penta D	13							
TH01	9.3				9.3			
vWA	19	20			19	20		
D21S11	30	30.2						
D7S820	9	11			9	11		
D5S818	10				10			
DYS391								
TPOX	8				8			
D8S1179	14							
D12S391	21	23						
D19S433	13	16.2						
FGA	22	24						
D22S1045	11	17						

Number of shared alleles between sample and database profile:	13
Total number of alleles in the database profile:	13
Percent match between the submitted sample and the database profile:	100%

The allele match algorithm compares the loci highlighted in grey only (8 core loci plus amelogenin).

EXPLANATION OF TEST RESULTS

☐ The submitted sample profile is human, but not a match for any profile in the STR database.

☑ The submitted sample profile showed 80% to 100% match for the following ATCC human cell line(s) in the STR database (8 core loci plus Amelogenin): **WI-38 VA-13 Subline 2RA Lung Human**

☐ The submitted profile is similar to the following ATCC human cell line(s):

☐ The submitted sample is a mixture. Multiple peaks are observed in the STR profiling results.

ADDITIONAL INFORMATION: Comparative Data Output from ATCC STR Profile Database

% Match	ATCC Number	Designation	D5S818	D13S317	D7S820	D16S539	vWA	TH01	AMEL	TPOX	CSF1PO
100	CCL-75.1	WI-38 VA-13 Subline 2RA Lung Human	10	11	9,11	11,12	19,20	9.3	X	8	10,12

For alternate database, you may visit https://www.dsmz.de/services/services-human-and-animal-cell-lines/online-str-analysis.html

End of report

Fragment Analysis
- STR Profiling
Cell Line Authentication

Axil Scientific Pte Ltd
2 Tukang Innovation Grove, #06-01, JTC MedTech Hub, Singapore 618305
T: +65 6775 7318
F: +65 6775 7211
E: sequencing@axilscientific.com

Apical Scientific Sdn Bhd
Lot 7-1 to 7-4, Jalan SP 2/7, Taman Serdang Perdana, Seksyen 2, 43300 Seri Kembangan, Selangor, Malaysia
T: +603 8943 3252
F: +603 8943 3243
E: sequencing@apicalscientific.com

CUSTOMER INFORMATION

Name	Poon Lai Fong	Order ID	892
Address		Date Sample Received	11-Nov-2022
Email	gmsplf@nus.edu.sg; laifong.poon@duke-nus.edu.sg	Report Date	22-Nov-2022

METHODOLOGY

Twenty-four short tandem repeat (STR) loci plus the gender determining locus, Amelogenin, were amplified using the commercially available GenePrint® 24 System from Promega. The sample was processed using the Applied Biosystems™ DNA Analyzer. Data were analyzed using GeneMapper® v4.0 software (Applied Biosystems™). Appropriate positive and negative controls were run and confirmed for each sample submitted.

SAMPLE INFORMATION

**Sample Name**	**Cell Line Designation**
U2OS	U-2-OS ATCC #HTB-96

STR PROFILING RESULTS

	Test Result for Sample				ATCC Reference Database Profile			
**LOCI**	**U2OS**				**U-2 OSOsteosarcomaHuman**			
Amelogenin	X				X			
D3S1358	11	16						
D1S1656	16	17.3						
D2S441	10	14						
D10S1248	13	14						
D13S317	13				13			
Penta E	10	13						
D16S539	11	12			11	12		
D18S51	12	14						
D2S1338	20	24						
CSF1PO	13				13			
Penta D	9							
TH01	6	9.3			6	9.3		
vWA	14	18			14	18		
D21S11	31							
D7S820	11	12			11	12		
D5S818	11				11			
DYS391								
TPOX	11	12			11	12		
D8S1179	12	14						
D12S391	19	20						
D19S433	15	14						
FGA	20							
D22S1045	15	16						

Number of shared alleles between sample and database profile:	14
Total number of alleles in the database profile:	14
Percent match between the submitted sample and the database profile:	100%

The allele match algorithm compares the loci highlighted in grey only (8 core loci plus amelogenin).

EXPLANATION OF TEST RESULTS

☐ The submitted sample profile is human, but not a match for any profile in the STR database.

☑ The submitted sample profile showed 80% to 100% match for the following ATCC human cell line(s) in the STR database (8 core loci plus Amelogenin): **U-2 OSOsteosarcomaHuman**

☐ The submitted profile is similar to the following ATCC human cell line(s):

☐ The submitted sample is a mixture. Multiple peaks are observed in the STR profiling results.

ADDITIONAL INFORMATION: Comparative Data Output from ATCC STR Profile Database

% Match	ATCC Number	Designation	D5S818	D13S317	D7S820	D16S539	vWA	TH01	AMEL	TPOX	CSF1PO
100	HTB-96	U-2 OSOsteosarcomaHuman	11	13	11,12	11,12	14,18	6,9.3	X	11,12	13
100	HTB-96-LUC2	U-2 OS-Luc2 Osteosarcoma Human	11	13	11,12	11,12	14,18	6,9.3	X	11,12	13

For alternate database, you may visit https://www.dsmz.de/services/services-human-and-animal-cell-lines/online-str-analysis.html

End of report
