# Supplementary figures and images for: High-throughput telomere length measurement at nucleotide resolution using the PacBio high fidelity sequencing platform
Source: Nat Commun. 2023 Jan 17;14:281. doi: 10.1038/s41467-023-35823-7 (PMC9845338; doi:10.1038/s41467-023-35823-7)

Uncropped teloblot raw data for Figure 1b

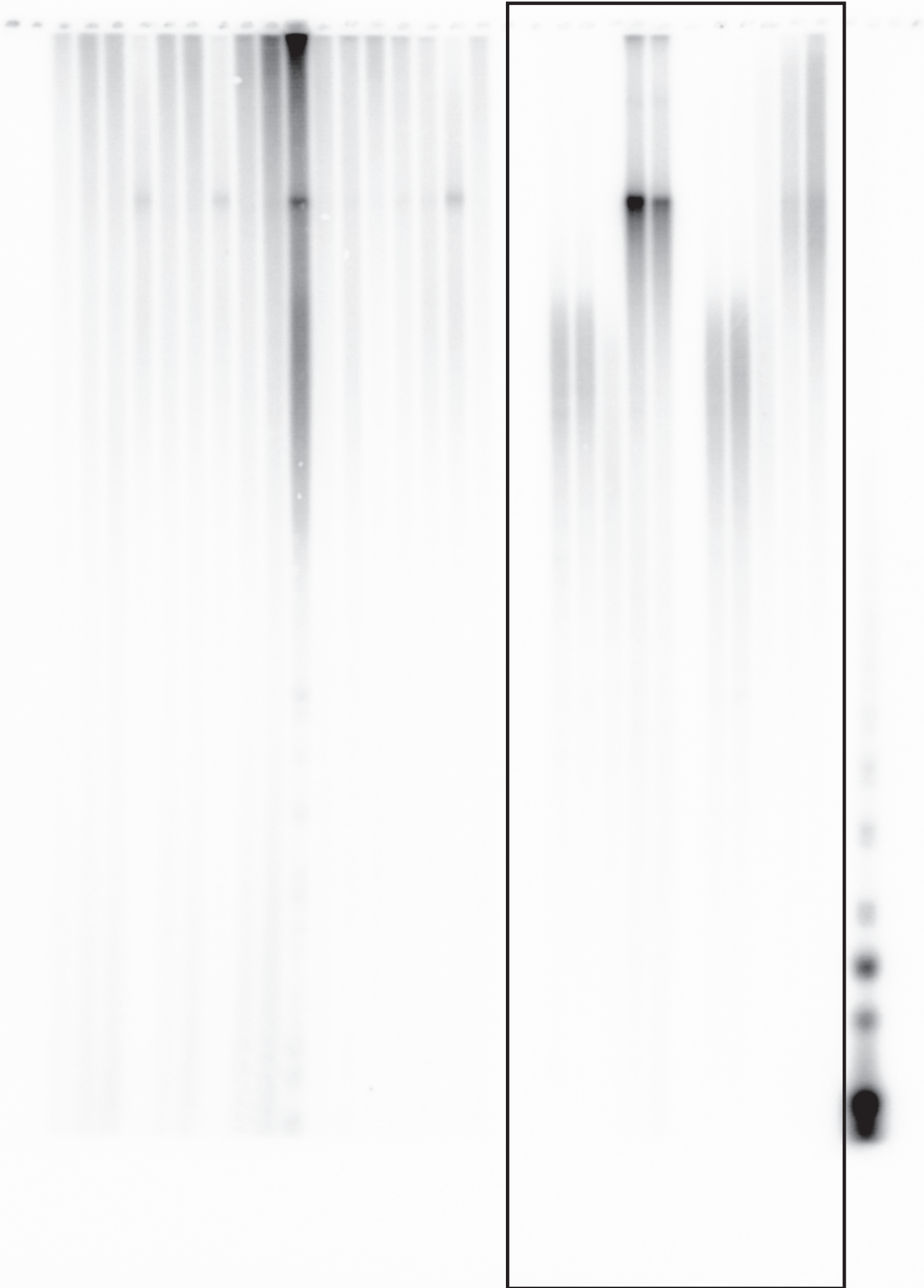

Uncropped teloblot raw data for Figure 2g

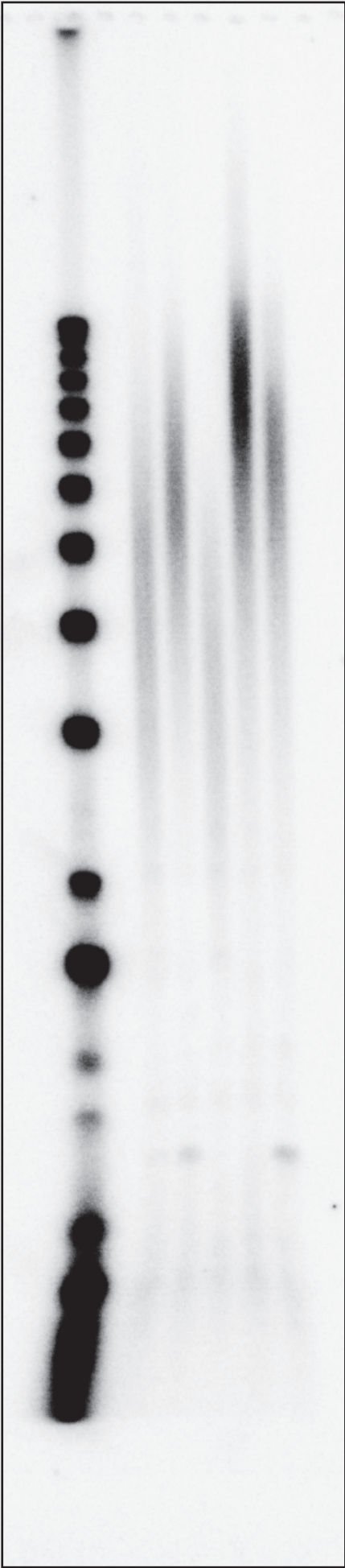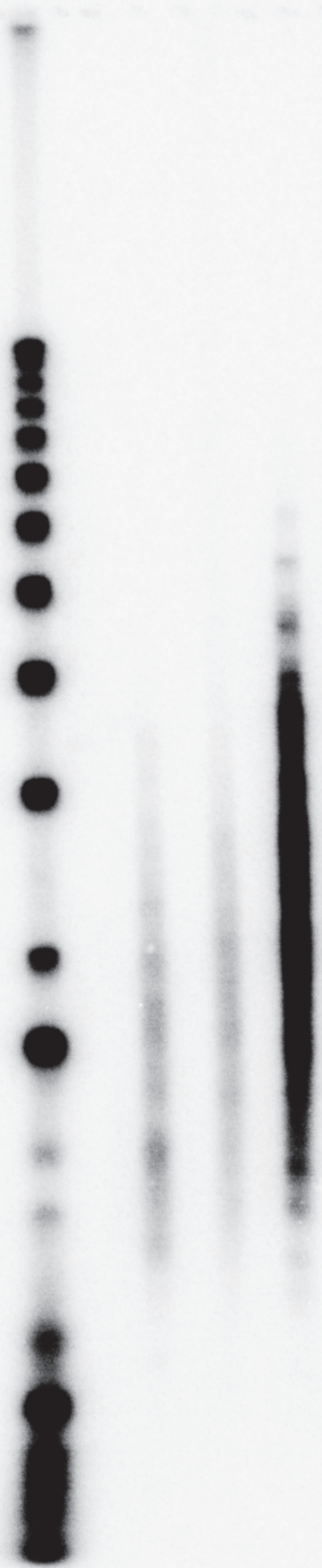

Supplement: Supplementary file 10 — Source data [file 41467_2023_35823_MOESM10_ESM.zip › Sourece data 1.pdf]
